# Supplementary material for: Exploring the barriers to, and importance of, participant diversity in early-phase clinical trials: an interview-based qualitative study of professionals and patient and public representatives
Source: BMJ Open. 2024 Mar 19;14(3):e075547. doi: 10.1136/bmjopen-2023-075547 (PMC10952868; doi:10.1136/bmjopen-2023-075547)
Supplement: Supplementary data [file bmjopen-2023-075547supp001.pdf]

EDI in early phase trials – qualitative interview topic guide (patients and research participants)

NOTES: This topic guide is a flexible tool and may be revised as new areas of interest arise during the process of data collection. The wording of questions is for guidance only and can be varied to suit the natural style of the interviewer and the level of understanding of the participant.

1. Welcome and context setting:

- Introduce yourself
- Guide the participant through the Participant Information Sheet
- "The interview will last approximately 45 to 60 minutes"
- Explain that, "to help us with this study, we would like to make a recording of what we all say today, but nobody will be able to identify you from that recording other than me. Is that okay?"
- Guide the participant through the consent form
- "You are free to withdraw at any point during the interview and you don't have to answer any of the questions if you don't want to, but once the interview has finished I will be unable to remove your information from the study, if you chose to withdraw"
- Check they are happy to continue and ask if there are any questions.

2. Describe to the interviewee the definitions of underserved groups and EDI (in lay person language) we are using (these will also be provided to participants to refer to):

- Remind participant why we are doing this research
- Give brief definition of "EDI"
- Give brief definition of an early phase trial

3. Questions

QUESTION 1: What do you understand of early phase trials?

PROMPT: Have you been approached to take part in such a study?

QUESTION 2: What is your experience of the process of being approached to take part, or taking part in, early phase trials?

PROMPT: How were you approached to take part?
PROMPT: How did you find being approached?
PROMPT: How did you make the decision as to whether or not to take part?
PROMPT: What factors influenced your decision to either participate, or not participate?

QUESTION 3: What is your experience of taking part (or being approached to take part in) early phase trials?

PROMPT: What challenges were there to you participating in early phase trials?

Qualitative interview topic guide (patients and research participants) V1.0 dated 23/03/2022

Cultural and institutional barriers, attitudes and beliefs, emotional and psychological barriers, financial barriers

PROMPT: Were there any enablers?
PROMPT: What challenges are there for others who are from underserved groups?
PROMPT: anything else you would like to mention?

QUESTION 4: What influences your decision regarding whether or not to participate in early phase trials?

QUESTION 5: What enables you to take part in early phase trials?

PROMPT: What challenges are there to you participating in early phase trials?
PROMPT: What challenges are there for others who are from underserved groups?

QUESTION 6: What benefits are there to taking part in these trials?

PROMPT: What benefits are there to you participating in early phase trials?
PROMPT: What benefits are there for others who are from underserved groups?

QUESTION 7: What can be done to encourage more people to participate in early phase trials?

Qualitative interview topic guide (patients and research participants) V1.0 dated 23/03/2022
