# Supplementary material for: Exploring the barriers to, and importance of, participant diversity in early-phase clinical trials: an interview-based qualitative study of professionals and patient and public representatives
Source: BMJ Open. 2024 Mar 19;14(3):e075547. doi: 10.1136/bmjopen-2023-075547 (PMC10952868; doi:10.1136/bmjopen-2023-075547)
Supplement: Supplementary data [file bmjopen-2023-075547supp002.pdf]

# EDI in early phase trials – qualitative interview topic guide

**NOTES:** This topic guide is a flexible tool and may be revised as new areas of interest arise during the process of data collection. The wording of questions is for guidance only and can be varied to suit the natural style of the interviewer and the level of understanding of the participant.

## 1. Welcome and context setting:

- Introduce yourself
- Remind the participant of the aims of the study
- Guide the participant through the Participant Information Sheet
- "The interview will last approximately 45 to 60 minutes"
- Explain that, "to help us with this study, we would like to make a recording of what we all say today, but nobody will be able to identify you from that recording other than me. Is that okay?"
- Guide the participant through the consent form
- "You are free to withdraw at any point during the interview and you don't have to answer any of the questions if you don't want to, but once the interview has finished I will be unable to remove your information from the study, if you chose to withdraw"
- Check they are happy to continue and ask if there are any questions.

*Remind the participants of the definition of underserved groups:*

We are using the National Institute for Health Research's (NIHR's) guidance from the INnovations in CLinical trial design and delivery for the UnDEr-served (INCLUDE) project to define an underserved group, which states that:

> "The work of the NIHR-INCLUDE project shows that there is no single definition for an under-served group. Some key characteristics that are common to several under-served groups are:
>
> - Lower inclusion in research than one would expect from population estimates
> - High healthcare burden that is not matched by the volume of research designed for the group
> - Important differences in how a group responds to or engages with healthcare interventions compared to other groups, with research neglecting to address these factors
>
> "A key finding from our work is that the definition of 'under-served' is often very context and study specific. An under-served group for one disease or type of study may be the opposite to that of another."

**EDI may be important due to:**

- Lower inclusion of underserved groups in research than one would expect from population estimates
- High healthcare burden that is not matched by the volume of research designed for the group

Qualitative interview topic guide V1.0 dated 23/03/2022

- Important differences in how a group responds to or engages with healthcare interventions compared to other groups, with research neglecting to address these factors

## 2. Questions (remind participant to give examples, where appropriate)

**QUESTION 1:** Generally, what do you think is the value of including underserved groups in early phase trials?

PROMPT: Is there any value to including underserved groups in early phase trials?

PROMPT: Should early phase trials provide equal access to underserved groups (diversity)?

PROMPT: Should early phase trials recognise differences between individuals?

PROMPT: What are the positive consequences?

PROMPT: What are the negative consequences?

PROMPT: Is the value of underserved groups in early phase different from late phase and if so how?

**QUESTION 2**: When may the inclusion of underserved groups in early phase trials be warranted, and unwarranted (with reasons)?

PROMPT: Are there any situations (e.g. diseases, treatments) where underserved groups is required? If so, why?

PROMPT: Are there any situations (e.g. diseases, treatments) where underserved groups is less important? If so, why?

**QUESTION 3:** What is your view on the outcome of including underserved groups in early phase trials?

PROMPT: Does including (or not including) underserved groups affect future, later phase trials?

PROMPT: Does including (or not including) underserved groups affect the regulatory process?

**QUESTION 4:** Have you incorporated underserved groups into any early phase trials you have conducted?

PROMPT: Did you specifically plan any strategies to include individuals from underserved groups? Which groups?

PROMPT: Why did you (or why didn't you) look to include underserved groups in the trial?

**QUESTION 5:** How did you (OR HOW WOULD YOU) incorporate underserved groups into previous early phase trials?

PROMPT: How did you identify and approach those from underserved groups?

PROMPT: Were there any other design considerations taken into account? E.g. translation, reducing burden, amending exclusion criteria.

Qualitative interview topic guide V1.0 dated 23/03/2022

**QUESTION 6** What are the challenges of including underserved groups in early phase trials? How have you, or would you, overcome these?

PROMPT: Have you encountered any challenges when incorporating underserved groups?

PROMPT: How did you, or might you overcome these challenges?

(if not already mentioned): Are there any challenges with regards to incorporating multiple underserved groups in a small early phase trial?

**QUESTION 7:** What enables (or helps) the incorporation of underserved groups into early phase trials?

## Describe the definitions of underserved groups and EDI we are using (these will also be provided to participants to refer to). Ask participant to reflect on these definitions:

### *EDI:*

**Equality** – Individuals are not treated less favourably due to their characteristics and have equal opportunities to take part in early phase trials, and are provided with equal opportunities if they decide to take part in a trial.

QUESTION: What are your reflections on this definition? Would you change it in any way?

QUESTION: What does equality mean in the context of early phase trials?

QUESTION: Do you think early phase trials provide equal access to groups (and do you think they should)?

QUESTION: Do you think people from particular groups are excluded from early phase trials?

**Diversity** – Recognising and respecting differences in individuals who are approached to take part, and decide to take part, in early phase trials.

QUESTION: What are your reflections on this definition? Would you change it in any way?

QUESTION: What does diversity mean in the context of early phase trials?

QUESTION: Do you think early phase trials recognise and respect the differences of individuals? Do you think they should?

**Inclusion** – Creating an environment where everyone feels welcomed and valued.

QUESTION: What does inclusion mean in the context of early phase trials?

QUESTION: Do you think early phase trials are inclusive? Do you think they should be?

Qualitative interview topic guide V1.0 dated 23/03/2022
