# Supplementary material for: Exploring the barriers to, and importance of, participant diversity in early-phase clinical trials: an interview-based qualitative study of professionals and patient and public representatives
Source: BMJ Open. 2024 Mar 19;14(3):e075547. doi: 10.1136/bmjopen-2023-075547 (PMC10952868; doi:10.1136/bmjopen-2023-075547)
Supplement: Author's manuscript [file bmjopen-2023-075547.draft_revisions.pdf]

BMJ Open is committed to open peer review. As part of this commitment we make the peer review history of every article we publish publicly available.

When an article is published we post the peer reviewers' comments and the authors' responses online. We also post the versions of the paper that were used during peer review. These are the versions that the peer review comments apply to.

The versions of the paper that follow are the versions that were submitted during the peer review process. They are not the versions of record or the final published versions. They should not be cited or distributed as the published version of this manuscript.

BMJ Open is an open access journal and the full, final, typeset and author-corrected version of record of the manuscript is available on our site with no access controls, subscription charges or pay-per-view fees (http://bmjopen.bmj.com).

If you have any questions on BMJ Open's open peer review process please email info.bmjopen@bmj.com

BMJ Open

A qualitative interview study exploring the barriers and importance of participant diversity in early phase clinical trials

Journal:	*BMJ Open*
Manuscript ID	bmjopen-2023-075547
Article Type:	Original research
Date Submitted by the Author:	11-May-2023

Complete List of Authors:	Chatters, Robin; The University of Sheffield, Sheffield Clinical Trials Research Unit (CTRU)<br>Dimairo, Munyaradzi; University of Sheffield, School of Health and Related Research<br>Cooper, Cindy; The University of Sheffield, Sheffield Clinical Trials Research Unit (CTRU)<br>Ditta, Shamila; The University of Sheffield, Sheffield Clinical Trials Research Unit (CTRU)<br>Woodward, Jonathan; The University of Sheffield, Sheffield Clinical Trials Research Unit (CTRU)<br>Biggs, Katie; The University of Sheffield, Sheffield Clinical Trials Research Unit (CTRU)<br>Ogunleye, Della; n/a - patient representative<br>Thistlewaite, Fiona; Christie NHS Foundation Trust and University of Manchester<br>Yap, Christina; Institute of Cancer Research<br>Rothman, Alexander; Sheffield Teaching Hospitals NHS Foundation Trust, Department of Infection, Immunity, and Cardiovascular Disease

Keywords:	Health Equity, QUALITATIVE RESEARCH, Clinical trials < THERAPEUTICS

SCHOLARONE™ Manuscripts

I, the Submitting Author has the right to grant and does grant on behalf of all authors of the Work (as defined in the below author licence), an exclusive licence and/or a non-exclusive licence for contributions from authors who are: i) UK Crown employees; ii) where BMJ has agreed a CC-BY licence shall apply, and/or iii) in accordance with the terms applicable for US Federal Government officers or employees acting as part of their official duties; on a worldwide, perpetual, irrevocable, royalty-free basis to BMJ Publishing Group Ltd ("BMJ") its licensees and where the relevant Journal is co-owned by BMJ to the co-owners of the Journal, to publish the Work in this journal and any other BMJ products and to exploit all rights, as set out in our licence.

The Submitting Author accepts and understands that any supply made under these terms is made by BMJ to the Submitting Author unless you are acting as an employee on behalf of your employer or a postgraduate student of an affiliated institution which is paying any applicable article publishing charge ("APC") for Open Access articles. Where the Submitting Author wishes to make the Work available on an Open Access basis (and intends to pay the relevant APC), the terms of reuse of such Open Access shall be governed by a Creative Commons licence – details of these licences and which Creative Commons licence will apply to this Work are set out in our licence referred to above.

Other than as permitted in any relevant BMJ Author's Self Archiving Policies, I confirm this Work has not been accepted for publication elsewhere, is not being considered for publication elsewhere and does not duplicate material already published. I confirm all authors consent to publication of this Work and authorise the granting of this licence.

For peer review only

A qualitative interview study exploring the barriers and importance of participant diversity in early phase clinical trials

Robin Chatters (r.chatters@sheffield.ac.uk) , Sheffield Clinical Trials Research Unit, School of Health and Related Research, The University of Sheffield, Regent Court, Regent Street, S1 4DA (Corresponding author).

Munya Dimairo (m.dimairo@sheffield.ac.uk), Sheffield Clinical Trials Research Unit, School of Health and Related Research, The University of Sheffield, Regent Street, S1 4DA.

Cindy Cooper (c.l.cooper@sheffield.ac.uk), Sheffield Clinical Trials Research Unit, School of Health and Related Research, The University of Sheffield, Regent Street, S1 4DA.

Shamila Ditta (s.ditta@sheffield.ac.uk), Sheffield Clinical Trials Research Unit, School of Health and Related Research, The University of Sheffield, Regent Street, S1 4DA.

Jonathan Woodward (jonathan.woodward@sheffield.ac.uk), Sheffield Clinical Trials Research Unit, School of Health and Related Research, The University of Sheffield, Regent Street, S1 4DA.

Katie Biggs (c.e.biggs@sheffield.ac.uk), Sheffield Clinical Trials Research Unit, School of Health and Related Research, The University of Sheffield, Regent Street, S1 4DA.

Della Ogunleye (ogunleyedella5@gmail.com) (patient representative), London, UK.

Fiona Thistlethwaite (fiona.thistlethwaite@nhs.net), The Christie NHS Foundation Trust and University of Manchester, Wilmslow Road, M20 4BX

Christina Yap (christina.yap@icr.ac.uk), Clinical Trials and Statistics Unit, Institute of Cancer Research, Sutton, Cotswold Road, SM2 5NG

Alexander Rothman (a.rothman@sheffield.ac.uk), Department of Infection, Immunity, and Cardiovascular Disease, The Medical School, The University of Sheffield and Sheffield Teaching Hospitals NHS Foundation Trust

Keywords: Equality, diversity, inclusion, early phase trial, qualitative

Word count: 5222

For peer review only - http://bmjopen.bmj.com/site/about/guidelines.xhtml

Abstract

Objectives: To explore the importance of, and barriers to achieving, diversity in early phase clinical trials.

Design: In-depth individual qualitative interviews; data analysed using thematic analysis.

Participants: Five professionals (clinical researchers, methodologists with knowledge of early phase clinical trials) and three patient and public representatives (those with experience of early phase clinical trials and/or those from ethnic minority backgrounds).

Results: Professionals viewed that diversity is not currently considered in all early phase clinical trials but felt that it should always be taken into account. Such trials are primarily undertaken at a very small number of centres, thus limiting the populations they can access. Referrals from clinicians based in the community may increase diversity, however, those referred are often not from underserved groups. Referrals may be hindered by the extra resources required to approach and recruit underserved groups and participants often having to undertake "self-driven" referrals. Patient and public representatives stated that diversity is important in research staff too and that potential participants should be informed of the need for diversity in clinical trials. Those from underserved groups may require clarification regarding the potential harms of the treatment, even if these are not known. Education may be key to improving awareness and perception of early phase clinical trials. We provide fourteen recommendations for researchers and funders to improve diversity in early phase clinical trials.

Conclusions: Diversity is important in early phase trials and should be considered in all trials. Consideration is required regarding the extent of diversity and how it is addressed. The increased resources needed to recruit those from underserved groups may warrant funders to increase the funds to support the recruitment of such participants. The potential harms and societal benefits of the research should be presented to potential participants in a balanced but accurate way to increase transparency.

Strengths and limitations of this study

- We qualitatively interviewed a diverse sample of five professionals (clinical researchers and methodologists) and three patient representatives to explore the importance of, and barriers to achieving, diversity in early phase clinical trials.

For peer review only - http://bmjopen.bmj.com/site/about/guidelines.xhtml

- Data saturation was not considered; rather, we looked to achieve 'information power', as conceived by Malterud *et al.* [28], where the size of the study was determined by the amount of information the sample holds.
- Professional interviewees were representatives from the NHS and UK Universities. All except one interviewee's experience was primarily in UK-based cancer early phase clinical trials. Therefore, the conclusions that can be made regarding other disease areas, sectors and nations are limited.

Background

The importance of equality, diversity and inclusion in clinical research is well documented, with the COVID-19 pandemic bringing into sharp focus the lack of inclusion of underserved groups in clinical trials [1–5]. The value of inclusion of such populations in late phase trials (i.e., in phases 3 and 4) is widely accepted, as treatments may affect diverse subsets of the population differently [6]. As such, there is guidance for researchers to consider groups routinely underserved in research when designing and conducting clinical trials [7–9]. There is evidence to suggest that diversity in such trials may be increased by improving staff competency through training and increased recruitment of staff from diverse backgrounds [6].

However, we are not aware of any guidance regarding the importance of diversity and how to incorporate underserved groups into early phase clinical trials (EPCTs). EPCTs are conducted in healthy volunteers or participants with a specific health condition and help us understand the feasibility of the therapeutic approach, the appropriate dose of the new treatment, how the drug should be administered, how the body responds to the treatment, and side effects. More so, they hugely inform the design and conduct of related later phase trials. Due to their small size and often high-risk nature, EPCTs have specific challenges regarding the inclusion of diverse populations. Despite the unknowns, the current lack of diversity in EPCTs is clear, with research suggesting that EPCTs underrepresent women [10], older adults [11–13], those with lower education levels [14], those who live further away from hospitals [14], those from deprived areas [15], and ethnic minorities [16,17]. It has been shown that participants experiencing socioeconomic deprivation have worse outcomes in cancer trials than those not experiencing socioeconomic deprivation [18], however, we do not know if this is the case for EPCTs due to the lack of data on diversity.

Unfortunately, there is a lack of interventions to address the inequalities within EPCTs [17]. Several studies have been undertaken to assess the barriers to participating in EPCTs, which include the information provided to potential participants [19], expectations of a therapeutic benefit from the

For peer review only - http://bmjopen.bmj.com/site/about/guidelines.xhtml

trial treatment [20,21], lack of social and financial support, [22] and restrictive eligibility criteria [23]. However, these studies focus on general recruitment barriers, rather than those that prevent underserved populations from participating. Potential methods of increasing diversity include community outreach, increased diversity of staff, culturally tailored interventions, bilingual study teams [6,24,25], and helping elderly adults navigate barriers to enrolment [26].

Previous research has not discussed the importance of the inclusion of underserved populations in EPCTs, or when it may be important. It may be the case, for certain conditions, treatments, or contexts, that a focus on the representation of the population is less important and therefore diverse populations may not be required. For example, due to suspected homogeneity in how individuals may react to the treatment under investigation. However, excluding certain populations from such trials may lead to an inequitable health policy stacked against underrepresented subpopulations. There is also evidence of a potential 'trial effect' of improved outcomes, greater adherence to clinical guidelines and evidence-based practice of clinicians and institutions that take part in trials [27]. However, the consequences for patient health are uncertain.

We, therefore, undertook a qualitative study of professionals with knowledge of EPCTs (i.e., clinical researchers and methodologists) and patient and public representatives to explore in which situations diversity may be important in EPCTs, how diversity has been (and could be) incorporated into EPCTs, and the barriers to inclusion in such trials.

Methods

Scope and participant eligibility

We undertook semi-structured qualitative interviews with both professionals (i.e., clinical researchers and methodologists with knowledge of EPCTs) and patient and public representatives (those with experience of EPCTs and/or those from ethnic minority backgrounds). Interviews with professionals explored the level of importance of diversity in EPCTs and when it may be appropriate, their experience of incorporating diversity into EPCTs, and the challenges of doing so. Interviews with the patient and public representatives focussed on the challenges of participating in EPCTs, and methods to improve diversity in such trials.

Recruitment of interview participants

Potential participants were identified by either searching institutional webpages or registries for individuals that are involved in EPCTs (e.g., clinical trials units (CTUs) that are listed on the UK Clinical Research Collaboration (UKCRC) CTU website as having expertise in phase 1 studies); asking study

For peer review only - http://bmjopen.bmj.com/site/about/guidelines.xhtml

collaborators and other contacts for any individuals they are aware of who may be experienced in conducting EPCTs; and via social media (e.g., Twitter).

Potential participants were sent an email with a participant information sheet (PIS) inviting them to participate in the study. To identify a purposive selection of participants for the interview, individuals were asked to complete a short survey to express their interest in the study which was administered using Qualtrics. The questionnaire requested key demographic data including location, age, ethnicity (patient and public representatives only), experience (professional or patient and public representative), clinical speciality (professionals only), area of expertise (professionals only), and gender. At the end of the survey, respondents were able to indicate whether they were interested in participating in an interview.

Participants were selected purposefully from those that completed the survey, maximising variation in key characteristics (area of expertise, ethnicity for the patient and public representatives only, years of experience, and gender). Additional participants were approached via email for the interview without having to complete the survey first if they were thought to increase the diversity of the sample.

Research ethics and participant consent process

Consent to participate in the semi-structured interviews was obtained via a consent form, which was completed by the participant prior to the interview, and after completing the survey (if applicable). The participant signed the consent form using an electronic (typed, or image of their signature inserted into the form) signature. The form was then countersigned by the researcher, and a copy of the completed consent form was emailed back to the participant.

Sample size

Data saturation was not considered; rather, we looked to achieve 'information power', as conceived by Malterud *et al.* [28], where the size of the study was determined by the amount of information the sample holds, with studies encompassing narrow aims, dense specificity and strong dialogue having higher information power. We aimed to interview between eight and ten individuals.

Study design and data collection

The study used a phenomenological framework, as interviewees had direct experience of the phenomenon under study. Two semi-structured topic guides were used (one for professionals and another for the patient and public representatives). These were developed by RC, with the aims of the study used as a starting point. The topic guides were then reviewed by members of the study team, including DO (a patient and public representative), but they were not piloted. Interviews were

For peer review only - http://bmjopen.bmj.com/site/about/guidelines.xhtml

conducted by RC (a male Research Fellow with a BSc), JW (a male Research Assistant with an MSc) and SD (a female Research Assistant with an MA). All three interviewers had the experience of working on clinical trials within a CTU, had previous experience of qualitative interviewing, and had a general interest in EDI in clinical research. Repeat interviews were not carried out, and transcripts were not returned to the participant for comment or correction. There were no other individuals present at the interviews and interviews lasted from 42 to 62 minutes. Participants undertook the interviewees either whilst at work or home. Field notes were not taken.

All interviews were undertaken via Google Meet, with the audio and video from the interview recorded (with consent) using in-built functionality within the Google Meet platform and transcribed for in-depth analysis. Transcripts were anonymised before analysis.

Emphasis was placed on collecting detailed data from experienced participants. Participants did not provide feedback on the findings.

Relationships of interviewee and interviewers

For most interviewees, there was no prior relationship between the interviewees and the interviewers. However, interviewees would have been aware of the interviewer's interest in this research area. Two interviewees (one clinical researcher and one patient and public representative) were involved in the study as collaborators. They provided input into the study protocol and topic guides and were therefore more aware than the other participants of the background, rationale and aims of the study.

Patient and public involvement

DO (a patient representative with experience of participating in research studies) provided input into all aspects of the study, including the aims and objectives, semi-structured topic guides, and findings.

Analysis

Data were analysed using thematic analysis, as described by Braun and Clarke [29]. NVivo software (version 12) was used to manage the data. Analysis was undertaken by RC and JW using the following steps; familiarisation, coding, and identification of themes, with both researchers initially coding two transcripts, agreeing on the codes, and RC then coding the remaining transcripts. Themes were derived from the data. There were two separate coding trees for the two main participant types – professionals and patient and public representatives, with main themes including the barriers to incorporating diverse populations, the need for diversity in EPCTs, current practice and issues, thoughts on how to improve diversity, and any positive and negative effects of including diverse populations.

For peer review only - http://bmjopen.bmj.com/site/about/guidelines.xhtml

Results

Study flow and characteristics of interviewees

Sixty-eight individuals were approached to participate in the study. Two individuals stated they did not want to take part due to a lack of time and five because of a lack of experience of EPCTs as well as 53 who did not reply. Five professionals (based within the National Health Service (NHS) and at UK Universities), and three patient and public representatives were interviewed – their characteristics are presented in Table 1. All participants, except one patient and public representative, were based in the UK and had the experience of cancer EPCTs.

Table 1: The characteristics of interviewed participants

Participant ID	Gender	Ethnicity*	Age range (years)	Role	Years of experience of early phase trials⁺	Clinical area of experience
Participant 1	Male	Not collected	25- 34	Professional (Operational Lead at a CTU)	6-10	Cancer
Participant 2	Female	Not collected	45-54	Professional (Doctor)	>16	Cancer
Participant 3	Female	Black African	55-64	Patient and public representative	Not collected	Cancer
Participant 4	Female	Not collected	45-54	Professional (Statistician)	11-15	Cancer
Participant 5	Male	Not collected	35-44	Professional (Statistician)	6-10	Cancer
Participant 6	Female	British Chinese	65-74	Patient and public representative	Not collected	Cancer
Participant 7	Female	White Canadian	35-44	Patient and public representative	Not collected	Neurological
Participant 8	Female	Not collected	35-44	Professional (Nurse)	11-15	Cancer

* Ethnicity only collected for the patient and public representatives; CTU, clinical trials unit

For peer review only - http://bmjopen.bmj.com/site/about/guidelines.xhtml

+ only collected for professional participants

Views on current practice

Professional interviewees stated that they do not currently specifically focus on trying to recruit underserved groups to EPCTs.

> *"I don't think it's something we consciously do. We basically open it to everyone who can enter into the trial, but we do not actively go and look for underserved patients."* (Participant 4, professional)

Those that did recall conversations on increasing diversity described rudimentary discussions around ensuring variation in the location of sites in the UK (e.g., North or South), and providing translation services, rather than in-depth conversations around how to increase the diversity of their sample.

> *"I think most of the time [discussions around diversity] will be about geography. It will be about "We've got a centre in London and I think I know someone up in Manchester. That'll be good because then we've got somewhere up north."* (Participant 5, professional)

It was evident that there is a lack of diversity in EPCTs, with three professionals describing the demographics of those that participate in EPCTs as being generally white, educated, affluent, and young.

> "*We know from our work here that patients coming through to our phase one trials are usually from the largely white, largely middle-class population*." (Participant 2, professional)

Participants felt that awareness is increasing, with three interviewees discussing studies they are aware of looking at the level of diversity in EPCTs, and another participant recently becoming aware of a funder requesting information about diversity in grant applications.

> "*The [EPCT] grant application I'm doing at the moment has three questions on [diversity], which we never had before. So, we're having to think about how we are going to address some of these issues*." (Participant 1, professional)

The level of importance for diversity in EPCTs

All interviewees stated that the inclusion of underserved groups is important and should always be considered when undertaking EPCTs.

> "*You'd never want to be ignoring [diversity in EPCTs], I don't think. Trying to think on the spot why you would – I can't think of an argument why you'd not want to consider it.*" (Participant 1, professional)

For peer review only - http://bmjopen.bmj.com/site/about/guidelines.xhtml

Participants thought that there is a general need to increase diversity in EPCTs without specifically focusing on particular attributes, underserved groups, or by setting a quota for underserved groups in the protocol.

> *"I don't think you can start to tease out who you should and shouldn't exclude in a phase 1 trial, so we should include everyone."* (Participant 2, professional)
>
> *"I don't know if working on a quota system of, say, "We're recruiting 30 participants and we need, in this particular study, we need at least 5 to be black" or "We want a 50:50 split male and female". I don't know how practical that is in the early phase setting."* (Participant 5, professional)

There were no situations when ensuring diversity may not be important. Even when undertaking an EPCT in a specific genetic biomarker or disease (e.g., prostate cancer in men), it would still be important to seek diversity in other characteristics (e.g., socioeconomic status).

> *"[a] prostate cancer study obviously can only recruit males. If you wanted to look at an effect on a certain group or you did want to target a certain socioeconomic group then that could be an area where you'd target and exclude others."* (Participant 1, professional)

The need for diversity in EPCTs

Interviewees discussed diversity in EPCTs in terms of the need for exploring treatment signals in diverse populations, research equity, and informing robust design and conduct of later phase trials.

Exploring treatment signals in diverse populations

The most frequently mentioned reason for increasing diversity in EPCTs was to ensure that EPCTs identify side-effects, tolerability, and efficacy in diverse populations early, as different populations may react variably to the study treatment. Many interviewees stated the aim of including diverse populations is to get a "feel" for the toxicities or a "signal" of the efficacy in these populations.

> *"… in the early phase you're not going to get the statistics to demonstrate that this drug is what the additional toxicities are, or that a different dose should be given or anything like that. I think it's about getting a feel for what the broad categories of toxicities are and what the dose is."* (Participant 2, professional)

Research equity

A key reason for diversifying clinical trial populations is to improve fairness and to enable underserved groups to access novel treatments.

> *"It's not fair that there are marginalised groups that are missing out on an opportunity to*

For peer review only - http://bmjopen.bmj.com/site/about/guidelines.xhtml

explore clinical trials and it might be that it's not the right thing for them and that might be the conclusion, but I'd much rather that patient have that informed decision about that before they make that decision." (Participant 8, professional)

Incorporating diverse groups into EPCTs allows treatments to be identified that may only be efficacious in certain populations. Assuming that treatments that are not efficacious in a Caucasian population do not work for everyone is an injustice if the treatment in fact works in other populations.

"[If you undertake a] trial [of] a drug in a Caucasian population and it doesn't have any efficacy signals […] there's an injustice there for the underserved ethnic populations, where actually it could be an efficacious drug." (Participant 2, professional)

Informing robust design and conduct of later phase trials

Improving the design and conduct of future trials was given as a reason to improve diversity, with specific considerations including understanding adherence to the new treatment in other populations, understanding how to better collect trial outcomes across diverse populations, and identifying stratification variables for later phase trials.

Barriers to diversity and methods to improve diversity

Interviewees provided their views on the barriers to incorporating diversity, and associated methods to improve representation, across the following themes: the location of recruiting centres, recruitment practices, the extra resources needed to recruit underserved groups, participant perception (awareness and transparency), and a lack of information on diversity in EPCTs. A summary of the proposed barriers and associated resolutions is provided in Table 2.

Location of recruiting centres

A main barrier to recruiting diverse populations was seen as the location of centres that undertake EPCTs, which are usually located in the centre of major cities. This can result in only those individuals that can travel to the centre being able to participate in the trial. This is compounded by the intensive time commitment required from participants to participate in these trials (e.g., intensive follow-up schedules).

"I think the main challenge for early phase is actually the fact that it's a lot more intensive if you enter into a trial in the early phase setting, compared to later. If you look at the time schedule of how many times you have to go into clinics, there's a lot more of them in the early phase." (Participant 4, professional)

For peer review only - http://bmjopen.bmj.com/site/about/guidelines.xhtml

Two methods were suggested to improve access to the research site. Firstly, both professionals and patient and public representatives suggested that participants and their carers or companions may need to be remunerated for their time (i.e., lost earnings), subsistence, and travel costs.

Secondly, referrals may be made from community hospitals. However, referrals from such centres often do not result in the recruitment of those from underserved groups due to participants often having to initiate the discussion regarding participation in an EPCT. To do so, the participant requires courage and conviction to ask their doctor about the trial, which may be less likely to be undertaken by those from underserved groups.

> *"Most of our patients that come to our unit are self-driven referrals [and] are well educated, articulate, confident, and would question their consultant and are driven by their own research into clinical trials and what's available and are driving their referral, …But what it does mean is that I feel we are not getting a representative population to even consider a clinical trial*." (Participant 8, professional)

Unfortunately, these actions were felt to improve access to the research site, but not the issue of the time commitment required of participants, an issue to which interviewees did not provide a resolution.

> *"Even if you fund the travel, it's still difficult to have the time [to participate in an EPCT]."* (Participant 4, professional)

Recruitment practices and trial design

The trial's eligibility criteria may indirectly reduce the diversity of the trial sample, which may be resolved by a patient or diversity representative reviewing the criteria to check for any elements that may inadvertently exclude individuals.

> *"It might be that you're excluding people who have a certain risk of another particular condition, whether that's a problem with their heart or some other issue … but what you're actually doing is you're making it far more likely that black women would not be eligible for that study because they're more likely to have that particular condition. Prior to the trial we engaged with an EDI representative to think about how we make this project as inclusive as possible. These were the steps we took. Therefore, our inclusion and exclusion criteria now read this, instead of this."* (Participant 5, professional)

Patient and public representatives felt it was important for the participant to be able to ask questions during the recruitment process. However, one professional participant stated many

For peer review only - http://bmjopen.bmj.com/site/about/guidelines.xhtml

participants often do not feel able to do this, with clinicians also not encouraging questions due to a lack of time. One participant suggested that a sheet of prompts could be given to the participant to refer to during a consultation with their doctor to assist them in asking questions related to the EPCT.

> *"I developed a question prompt list for patients to have in their hand and they could then refer to the questions that I developed with patients about what they felt they perhaps should have asked at the beginning. it gave the patient permission to ask those questions, [for example] emotive questions like "Will this trial cure my cancer?"* (Participant 8, professional)

Extra resources needed to recruit underserved groups

Approaching underserved groups to participate in EPCTs was seen as time-consuming. The use of translators to help those without English as their first language to comprehend the trial was seen as a key issue, with this adding to the cost and time demands.

> *"Clinic appointments tend to take longer if there's an interpreter to make sure everything's been explained clearly and that the patient has a good understanding. And ultimately, if a clinic appointment takes longer and you have a high proportion of patients requiring interpreters for example, it potentially reduces the number of patients that you can recruit to a trial."* (Participant 2, professional)

These issues may be intensified in deprived areas due to increased staff turnover and the reliance on locum doctors who lack the time or training to undertake recruitment processes. The increased workload required to recruit those from underserved populations does not result in increased funding.

> *"Someone who's going to bring in patients from more diverse populations isn't going to generate any additional income, because we don't have a supply issue. And actually… [it] might decrease our income because patients who need a translator take longer in clinical time and might be able to treat fewer patients." (*Participant 2, professional*)*

Therefore, interviewees recommended changes that funders could make, including providing trial teams with extended trial recruitment phases to allow time to recruit those from underserved populations. Additionally, extra per-participant funding for recruiting those from underserved groups may be required.

> *"If you go down to the health economics of it, you've got to have a driver that will benefit us in some way and maybe that's some kind of funding from [the] NIHR, where if a patient is*

For peer review only

For peer review only - http://bmjopen.bmj.com/site/about/guidelines.xhtml

> *labelled as being from a hard-to-reach population, that patient attracts additional income."* (Participant 2, professional)

Awareness and perception of EPCTs

Interviewees suggested that the awareness of EPCTs could be increased. This could be achieved by providing potential participants with access to databases that allow participants to be identified. For example, using a similar portal to the Experimental Cancer Medicine Centres (ECMC) trial finder for cancer EPCTs (which is currently only open to clinicians). Conversations about recruitment to EPCTs should be initiated as early as possible (ideally by the general practitioner, GP). Patient advocacy groups could be used to assist in reaching out to underserved patient groups in a balanced manner.

> *"I think it also requires quite a strong patient advocacy group of some sort that currently I don't think exists for EPCTs, which I think you need to reach out for those people to kind of reach out to people who don't normally think about entering into early phase trials. Kind of highlighting pros and cons, being very balanced."* (Participant 4, professional)

Patient and public representatives focussed on their perception of EPCTs. The safety of participating in an EPCT was seen as one of the main barriers to the inclusion of underserved groups, with the perceived risk being high, which was potentially exacerbated by the feeling of being a "guinea pig".

> *"I think in the Chinese culture, and some others, they are normally equated as guinea pigs and you've got to be self-sacrificing, or prepared to be, before you think about something like this, something drastic might befall you. Which is rather scaremongering."* (Participant 6, patient and public representative)

Several professional and patient and public representatives proposed that education may improve awareness and perception of EPCTs, with suggestions including the provision of general health education in schools, educating underserved groups, and educating refers in the need for diversity within EPCTs. However, there was a lack of suggestions on how to achieve this.

> *"I think [education is] key. Educating referrers and somehow educating [underserved] groups about the potential for accessing clinical trials. And I don't know fully how that's going to happen, how we can do that effectively." (*Participant 8, professional*)*

Communicating the level of risk associated with participating in an EPCT was seen as key, with safety being a key concern for public representatives, who may want detailed information about what is already known about the drug.

Transparency was seen as being particularly important. Several methods of increasing transparency were suggested, including providing potential participants with testimonials from other participants

For peer review only - http://bmjopen.bmj.com/site/about/guidelines.xhtml

and describing the need for diversity to those from underserved groups (i.e., why they are seeking diverse populations to participate in the study, and how they are including such groups).

One participant suggested that researchers should be honest if there is a lack of evidence regarding the safety of a new treatment (e.g., a drug).

> *"If the researcher feels that "if I share with the potential participant how little actually I know, they will lose confidence." But I would argue that conversely, if you don't share with me what you know and what you don't know, and why am I involved, do I have any valid role in this? Can I actually make a contribution in some areas? So, in fact, transparency and that openness is confidence enforcing."* (Participant 6, patient and public representative)

The need for transparency also extended to what *is not likely* to happen to a participant in an EPCT. One patient and public representative described that underserved populations have a lack of awareness of EPCTs and may assume that there is a significant risk of death. Researchers should therefore clarify the likely adverse events the participant may experience, for example, clarifying that death or paralysis is very unlikely. This could take the form of describing how other people have reacted to the drug.

> "*Has somebody like me done it before, how did they react to it?... It's just reassuring me that it's gonna be okay, you know, you wouldn't be paralysed."* (Participant 3, patient and public representative).

A lack of diversity was seen as not only a problem in the research participants themselves, but also in the staff and researchers that undertake and run such studies.

> *"The more diverse the people conducting the clinical trials, the more diverse it is, the better you see people forthcoming. "Oh, yes, I want to be part of that" When you don't see people like you in it, you know, what effect would it have on me? So, I know we're getting there but, you know, we still need to do more."* (Participant 3, patient and public representative)

Lack of information on diversity in EPCTs

Professional participants suggested that little is currently known about the current level of diversity within EPCTs, and therefore, postcode data could be collected for all participants screened for entry into an EPCT to identify if trials are being accessed by underserved groups. However, there may be ethical challenges in collecting such data for those participants that do not consent to participate in the trial.

For peer review only - http://bmjopen.bmj.com/site/about/guidelines.xhtml

Discussion

In this qualitative study, we have identified that the diversity of underserved groups does not seem to be currently routinely considered in EPCTs, but it is always viewed as relevant. The main benefit of including diverse populations in EPCTs is the identification of early signals of harms and efficacy in diverse populations, including underserved subpopulations, and providing equitable opportunities for patients. Study teams should make a concerted effort to recruit those from underserved groups into their EPCT and should seek to minimise the numerous barriers that prevent such individuals from participating. The most notable barrier appeared to be that EPCTs are primarily undertaken at a small number of specialist centres, predominately located within the centre of large cities, thus limiting the populations they can access. To increase diversity, these centres may need to rely on referrals from "feeder" centres located in the community. However, the extra resources it requires to approach and recruit underserved populations (e.g., language translation) can prevent clinicians from referring them. Patient and public representatives stated that trust and communication are important – those recruiting underserved groups to EPCTs should be transparent regarding the need for diversity in clinical trials, the potential harms of the treatment, and if these are not known, clarifying the likelihood of severe illness to ease their concerns. Education of both patients/public and clinical staff was felt to be key to increasing awareness and perception of EPCTs.

The strengths of this study are that both professionals and patient and public representatives were sought to obtain the views of two of the key stakeholders involved in EPCTs. Both sets of individuals were diverse, in terms of the role of professional participants (including clinicians, statisticians, and operational leads) and the ethnicity of the patient and public representatives. Due to funding limitations, only a small number of interviews were undertaken with representatives from the NHS and UK Universities. Those from other organisations (e.g., the pharmaceutical industry) were not interviewed. Additionally, all except one interviewee's experience was primarily in UK-based cancer EPCTs, therefore limiting the conclusions that can be made regarding other disease areas and nations.

This study supports the literature regarding the need to increase diversity in clinical trials [6,30]. Some of the barriers and methods to increase diversity have been reported previously, including adequately funding participants' time and travel costs [31], informing community oncologists of the trial to aid recruitment [26], and translation and language issues [24]. We have identified that, due to time constraints, those who refer to clinical trials may not approach those from underserved groups, which has been found in a previous study [32]. Our study adds to this literature by clarifying that diversity is important in all EPCTs, and by identifying additional barriers that, as far as we are

For peer review only - http://bmjopen.bmj.com/site/about/guidelines.xhtml

aware, have not been previously reported. These additional barriers concern the effect of the location of EPCT units on their ability to access underserved groups and the lack of referrals to these units of underserved groups from referrers in the community. Such referrals may be hindered by the need for participants to “self-drive” the referral, which may be a barrier given the lack of knowledge of EPCTs in underserved groups. We have also identified specific issues related to transparency, including informing the participant the reason for the need to focus on the recruitment of underserved groups, and providing the participant with an indication of the level of risk associated with the EPCT, even if the effects of the drug are unknown.

This study implies that those designing and recruiting to EPCTs should attempt to increase the diversity of their sample by referring to the fourteen principles outlined in *Table 3*. In this table, the authors have supplemented the findings from the qualitative study with their own reflections, which are provided in *italics*.

It should be noted that given that those participating in EPCTs may have optimistic expectations of the effect of participating in an EPCT [21], highlighting the harms of such trials may have a negative effect on recruitment, so researchers may also describe to participants the potential societal benefits of EPCTs highlighted in this study (e.g., improving the equity of research, exploring early treatment signals in diverse populations, and informing the robust design of future trials). Future research should focus on identifying the barriers and enablers to recruitment in a larger diverse sample of participants and by investigating how potential harms are communicated to participants, especially in the absence of quantifiable data. Overcoming the inherent barriers to undertaking clinical trials in novel treatments, which, due to their risk, often require participants having to regularly attend the hospital for safety checks, may be challenging but would open up EPCTs to patient groups who are currently unable to participate in such trials. These “remote” EPCTs have been broached, but are rarely undertaken in practice [33]. Additionally, educational packages could be developed to increase awareness and improve the perception of EPCTs in underserved groups – future research may focus on the content, audience, timing, and format of such packages.

Declarations

Author contributorship

RC, MD and CC devised the study. RC, SD and JW collected the data and undertook analysis. RC wrote the first draft of the report. DO was a patient representative who provided input into the design of the study and the findings. All authors provided input into the oversight of the study, critically reviewed the draft, and provided approval for publication.

For peer review only - http://bmjopen.bmj.com/site/about/guidelines.xhtml

Competing interests

The authors declare that they have no competing interests.

Funding

The study was funded by Research England via the Participatory Research Fund.

Data sharing

The dataset is the in-depth interview transcripts. Access is restricted to the study team in accordance with the requirements of the ethical approval.

Patient consent

Participants provided informed consent to participate in the study and for their quotations to be anonymously published.

Ethical approval

The study received favourable ethics approval from the research ethics committee based at the School of Health and Related Research (ScHARR), within the University of Sheffield (reference number: 045712).

Acknowledgements

We would like to thank the eight participants for their time in participating in the interviews and for sharing their valuable knowledge with us.

References

1 Diversity Data Report. Published Online First: 2021.https://www.nihr.ac.uk/about-us/our-key-priorities/equality-diversity-and-inclusion/diversity-data-report.htm

2 UK government sets out bold vision for the future of clinical research delivery - GOV.UK. https://www.gov.uk/government/news/uk-government-sets-out-bold-vision-for-the-future-of-clinical-research-delivery (accessed 12 Jan 2022).

3 Inclusion of Women and Minorities as Participants in Research Involving Human Subjects | grants.nih.gov. https://grants.nih.gov/policy/inclusion/women-and-minorities.htm (accessed 12 Jan 2022).

4 Improving inclusion of under-served groups in clinical research: Guidance from INCLUDE project. https://www.nihr.ac.uk/documents/improving-inclusion-of-under-served-groups-in-clinical-research-guidance-from-include-project/25435 (accessed 12 Jan 2022).

For peer review only - http://bmjopen.bmj.com/site/about/guidelines.xhtml

5 UKRI equality diversity and inclusion strategy: draft for consultation – UKRI. https://www.ukri.org/publications/equality-diversity-and-inclusion-strategy-draft-for-consultation/ukri-equality-diversity-and-inclusion-strategy-draft-for-consultation/ (accessed 17 Jan 2022).

6 Bodicoat DH, Routen AC, Willis A, *et al.* Promoting inclusion in clinical trials—a rapid review of the literature and recommendations for action. *Trials* 2021;**22**. doi:10.1186/S13063-021-05849-7

7 Treweek S, Banister K, Bower P, *et al.* Developing the INCLUDE Ethnicity Framework—a tool to help trialists design trials that better reflect the communities they serve. *Trials* 2021;**22**:1–12. doi:10.1186/S13063-021-05276-8/TABLES/3

8 Jutlla K. INCREASING PARTICIPATION OF BLACK ASIAN AND MINORITY ETHNIC (BAME) GROUPS IN HEALTH AND SOCIAL CARE RESEARCH. 2018.

9 Equality Impact Assessment Guidance and Template. http://www.acas.org.uk/media/pdf/s/n/Acas_managers_guide_to_equality_assessments.pdf (accessed 31 Aug 2022).

10 Pinnow E, Sharma P, Parekh A, *et al.* Increasing Participation of Women in Early Phase Clinical Trials Approved by the FDA. *Women's Heal Issues* 2009;**19**:89–93. doi:10.1016/j.whi.2008.09.009

11 Baldini C, Charton E, Schultz E, *et al.* Access to early-phase clinical trials in older patients with cancer in France: the EGALICAN-2 study. *ESMO Open* 2022;**7**:100468. doi:10.1016/J.ESMOOP.2022.100468

12 Khan KH, Yap TA, Ring A, *et al.* Phase I trial outcomes in older patients with advanced solid tumours. *Br J Cancer* 2016;**114**:262–8. doi:10.1038/bjc.2015.477

13 Mahipal A, Denson AC, Djulbegovic B, *et al.* Effect of Age on Clinical Outcomes in Phase 1 Trial Participants. *Cancer Control* 2015;**22**:235–41. doi:10.1177/107327481502200217

14 Gad KT, Johansen C, Duun-Henriksen AK, *et al.* Socioeconomic differences in referral to phase i cancer clinical trials: A danish matched cancer case-control study. *J Clin Oncol* 2019;**37**:1111–9. doi:10.1200/JCO.18.01983

15 Mohd Noor A, Sarker D, Vizor S, *et al.* Effect of Patient Socioeconomic Status on Access to Early-Phase Cancer Trials. *J Clin Oncol* 2013;**31**:224–30. doi:10.1200/JCO.2012.45.0999

For peer review only - http://bmjopen.bmj.com/site/about/guidelines.xhtml

16 Dunlop HM, Fitzpatrick E, Kurti K, *et al.* Abstract PR-05: Worsening disparities of racial minority participation in phase 1 early drug development trials in the United States, 2001-2018. *Cancer Epidemiol Biomarkers Prev* 2022;**31**:PR-05-PR-05. doi:10.1158/1538-7755.DISP21-PR-05

17 Perni S, Moy B, Nipp RD. Disparities in phase 1 cancer clinical trial enrollment. *Cancer* 2021;**127**:4464–9. doi:10.1002/cncr.33853

18 Unger JM, Moseley AB, Cheung CK, *et al.* Persistent Disparity: Socioeconomic Deprivation and Cancer Outcomes in Patients Treated in Clinical Trials. *J Clin Oncol* 2021;**39**:1339–48. doi:10.1200/JCO.20.02602

19 Cox K. Informed consent and decision-making: patients' experiences of the process of recruitment to phases I and II anti-cancer drug trials. *Patient Educ Couns* 2002;**46**:31–8. doi:10.1016/s0738-3991(01)00147-1

20 Meropol NJ, Weinfurt KP, Burnett CB, *et al.* Perceptions of patients and physicians regarding phase I cancer clinical trials: implications for physician-patient communication. *J Clin Oncol* 2003;**21**:2589–96. doi:10.1200/JCO.2003.10.072

21 Catt S, Langridge C, Fallowfield L, *et al.* Reasons given by patients for participating, or not, in Phase 1 cancer trials. *Eur J Cancer* 2011;**47**:1490–7. doi:10.1016/j.ejca.2011.02.020

22 Castillo G, Lalu MM, Asad S, *et al.* Navigating choice in the face of uncertainty: using a theory informed qualitative approach to identifying potential patient barriers and enablers to participating in an early phase chimeric antigen receptor T (CAR-T) cell therapy trial. *BMJ Open* 2021;**11**:e043929. doi:10.1136/bmjopen-2020-043929

23 Ho J, Pond GR, Newman C, *et al.* Barriers in phase I cancer clinical trials referrals and enrollment: five-year experience at the Princess Margaret Hospital. *BMC Cancer* 2006;**6**:263. doi:10.1186/1471-2407-6-263

24 Chalela P. Promoting Factors and Barriers to Participation in Early Phase Clinical Trials: Patients Perspectives. *J Community Med Health Educ* 2014;**04**. doi:10.4172/2161-0711.1000281

25 Pham B, Earle N, Rabel K, *et al.* Maximizing the diversity of participants in a phase II clinical trial of optical technologies to detect cervical neoplasia. *Gynecol Oncol* 2007;**107**:S208–14. doi:10.1016/J.YGYNO.2007.07.063

For peer review only - http://bmjopen.bmj.com/site/about/guidelines.xhtml

26 Basche M, Barón AE, Eckhardt SG, *et al.* Barriers to Enrollment of Elderly Adults in Early-Phase Cancer Clinical Trials. *J Oncol Pract* 2008;**4**:162–8. doi:10.1200/JOP.0842001

27 Clarke M, Loudon K. Effects on patients of their healthcare practitioner's or institution's participation in clinical trials: a systematic review. *Trials* 2011;**12**:16. doi:10.1186/1745-6215-12-16

28 Malterud K, Siersma VD, Guassora AD. Sample Size in Qualitative Interview Studies. *Qual Health Res* 2016;**26**:1753–60. doi:10.1177/1049732315617444

29 Braun, V., Clark V. Using thematic analysis in psychology. *Qual Res Psychol* 2006;**3**:77–101.

30 Inclusion of Older Adults in Cancer Clinical Trials Guidance for Industry. https://www.fda.gov/media/156616/download#:~:text=The issue of older adults being underrepresented in,malignancy%2C all of which may increase with age.

31 Bierer BE, White SA, Gelinas L, *et al.* Fair payment and just benefits to enhance diversity in clinical research. *J Clin Transl Sci* 2021;**5**:e159. doi:10.1017/cts.2021.816

32 Gomes F, Descamps T, Lowe J, *et al.* Enrolment of older adults with cancer in early phase clinical trials—an observational study on the experience in the north west of England. *Age Ageing* 2021;**50**:1736–43. doi:10.1093/ageing/afab091

33 Alemayehu D, Hemmings R, Natarajan K, *et al.* Perspectives on Virtual (Remote) Clinical Trials as the "New Normal" to Accelerate Drug Development. *Clin Pharmacol Ther* 2022;**111**:373–81. doi:10.1002/cpt.2248

Table 2: Barriers and facilitators to increasing diversity in EPCTs

**Barrier**	**Facilitator**
Many centres that recruit to EPCTs are located in the centre of major cities which may be challenging for certain populations to access	• Referrals can be made from community hospitals<br>• Provide reimbursement to trial participants and their carers for travel and subsistence costs
EPCTs require intensive time commitment from trial participants, which may deter involvement	None discussed

For peer review only - http://bmjopen.bmj.com/site/about/guidelines.xhtml

Participants may have to undertake "self-driven" referrals and require the motivation and courage to initiate discussions regarding EPCTs with their clinician	• Educate potential participants regarding the EPCTs that are available to them<br>• Educate clinicians to enable them to approach and recruit participants to EPCTs
Eligibility criteria may inadvertently reduce the diversity of the trial sample	• Engage with a patient or diversity representative to review eligibility criteria
It is important for participants to ask questions about the EPCT when discussing their potential participation, but participants may not ask questions, and clinicians may not encourage them, due to a lack of time	• A list of potential questions can be given to participants to prompt them during a consultation with their doctor/clinician
Approaching underserved groups to participate in an EPCT requires extra resources	• Additional funding and time may be required to recruit those from underserved groups
There is a lack of awareness of EPCTs amongst underserved groups	• Discussions around participating in an EPCT should be initiated as early as possible, ideally by the GP<br>• Education to improve awareness, both for patients and clinical staff.<br>• Permit use of trial databases to allow participants to locate EPCTs (e.g., Experimental Cancer Medicine Centres (ECMC) in cancer).<br>• Patient advocacy groups could be established to assist in relaying information to underserved groups in a balanced manner.
Those from underserved groups may have concerns around the safety of participating in an EPCT	• Communicate the level of risk associated with the EPCT with the participant<br>• Provide detailed information about what is already known about study treatment

For peer review only - http://bmjopen.bmj.com/site/about/guidelines.xhtml

	• Provide detail as to what is unlikely to happen to participants (e.g., death, paralysis)<br>• Educate patients to help improve their perception of EPCTs.
A perceived lack of transparency may result in those from underserved groups being unlikely to participate in an EPCT	• Provide potential participants with testimonials from other participants<br>• Describe the need for diversity in the EPCT to those from underserved groups and the importance of participating
A lack of diversity in trial staff and researchers may deter participants from taking part	• Increase the diversity of staff that undertake and run EPCTs

Table 3: Recommendations for researchers and funders

**Recommendations for researchers**
1) Increasing the diversity of the sample should always be considered in EPCTs to a) explore treatment signals in diverse populations, b) improve research equity, and c) inform the robust design and conduct of later phase trials. *Some diseases have specific diversity profiles, which should be reflected in the trial sample.*
2) Those undertaking EPCTs should make a concerted effort to recruit those from underserved groups. The diversity of EPCTs should generally be increased, rather than focussing on specific attributes or quotas.
3) Important markers of diversity (e.g. postcode, *ethnicity, age*) should be consistently collected and report from all those that are screened to participate in EPCTs.
4) In order to reach diverse communities, potential participants may be referred from community hospitals. However, participants may still struggle to access the main trial site, so reimbursement for participants and their carers time and travel costs should be considered. *Enhancing participant awareness of the reimbursement of travel and other costs is important.*

For peer review only - http://bmjopen.bmj.com/site/about/guidelines.xhtml

5) Education of both participants and staff is important to ensure both parties are aware of EPCTs, in order to enable recruitment and retention.
6) Researchers should ensure eligibility criteria do not inadvertently exclude those from underserved groups and should seek input from patient or diversity representatives into these criteria. *However, this should be balanced with the risk of the EPCT – some exclusions may be justified based on safety concerns.*
7) A list of prompts may be provided to participants to refer to during conversations regarding participation in an EPCT to assist them in asking questions about the study.
8) Discussions around entry into an EPCT should commence as early as possible in the participant's journey, ideally with the GP.
9) Participants from underserved groups may be concerned about the risks of participating in an EPCT. In order to reassure them, researchers may:<br>a. Clearly communicate the level of risk associated with the EPCT with the participant. The societal benefits of participating in an EPCT may also be relayed to participants (see principle 1);<br>b. Provide detailed information about what is already known about the study treatment;<br>c. Provide an honest explanation of the amount available regarding the evidence regarding the effects of the study treatment;<br>d. Provide information as to what is *unlikely* to happen to participants as a result of the study treatment (e.g., death, paralysis).
10) In order to assist in relaying information to underserved groups in a balanced manner, patient advocacy groups may be formed.
11) Due to a perceived lack of transparency, those from underserved groups may be provided with testimonials from other participants. The need for diversity in the EPCT may be explained to such individuals.
12) The diversity of those staff that recruit to EPCT should be increased in order to not deter underserved groups from participating.
**Recommendations for funders**

For peer review only - http://bmjopen.bmj.com/site/about/guidelines.xhtml

13) Funders may need to provide additional per participant funds for the recruitment of those from underserved groups due to the additional resources required to recruit such individuals.
14) Funders should request details of the methods investigators will use to identify, approach and recruit those from underserved groups in grant applications.

For peer review only

For peer review only - http://bmjopen.bmj.com/site/about/guidelines.xhtml

COREQ (COnsolidated criteria for REporting Qualitative research) Checklist

A checklist of items that should be included in reports of qualitative research. You must report the page number in your manuscript where you consider each of the items listed in this checklist. If you have not included this information, either revise your manuscript accordingly before submitting or note N/A.

Topic	Item No.	Guide Questions/Description	Reported on Page No.
**Domain 1: Research team and reflexivity**			
Personal characteristics			
Interviewer/facilitator	1	Which author/s conducted the interview or focus group?	
Credentials	2	What were the researcher's credentials? E.g. PhD, MD	
Occupation	3	What was their occupation at the time of the study?	
Gender	4	Was the researcher male or female?	
Experience and training	5	What experience or training did the researcher have?	
Relationship with participants			
Relationship established	6	Was a relationship established prior to study commencement?	
Participant knowledge of the interviewer	7	What did the participants know about the researcher? e.g. personal goals, reasons for doing the research	
Interviewer characteristics	8	What characteristics were reported about the inter viewer/facilitator? e.g. Bias, assumptions, reasons and interests in the research topic	
**Domain 2: Study design**			
Theoretical framework			
Methodological orientation and Theory	9	What methodological orientation was stated to underpin the study? e.g. grounded theory, discourse analysis, ethnography, phenomenology, content analysis	
Participant selection			
Sampling	10	How were participants selected? e.g. purposive, convenience, consecutive, snowball	
Method of approach	11	How were participants approached? e.g. face-to-face, telephone, mail, email	
Sample size	12	How many participants were in the study?	
Non-participation	13	How many people refused to participate or dropped out? Reasons?	
Setting			
Setting of data collection	14	Where was the data collected? e.g. home, clinic, workplace	
Presence of non-participants	15	Was anyone else present besides the participants and researchers?	
Description of sample	16	What are the important characteristics of the sample? e.g. demographic data, date	
Data collection			
Interview guide	17	Were questions, prompts, guides provided by the authors? Was it pilot tested?	
Repeat interviews	18	Were repeat inter views carried out? If yes, how many?	
Audio/visual recording	19	Did the research use audio or visual recording to collect the data?	
Field notes	20	Were field notes made during and/or after the inter view or focus group?	
Duration	21	What was the duration of the inter views or focus group?	
Data saturation	22	Was data saturation discussed?	
Transcripts returned	23	Were transcripts returned to participants for comment and/or	

For peer review only

Topic	Item No.	Guide Questions/Description	Reported on Page No.
		correction?	
**Domain 3: analysis and findings**			
Data analysis			
Number of data coders	24	How many data coders coded the data?	
Description of the coding tree	25	Did authors provide a description of the coding tree?	
Derivation of themes	26	Were themes identified in advance or derived from the data?	
Software	27	What software, if applicable, was used to manage the data?	
Participant checking	28	Did participants provide feedback on the findings?	
Reporting			
Quotations presented	29	Were participant quotations presented to illustrate the themes/findings? Was each quotation identified? e.g. participant number	
Data and findings consistent	30	Was there consistency between the data presented and the findings?	
Clarity of major themes	31	Were major themes clearly presented in the findings?	
Clarity of minor themes	32	Is there a description of diverse cases or discussion of minor themes?	

Developed from: Tong A, Sainsbury P, Craig J. Consolidated criteria for reporting qualitative research (COREQ): a 32-item checklist for interviews and focus groups. *International Journal for Quality in Health Care*. 2007. Volume 19, Number 6: pp. 349 – 357

Once you have completed this checklist, please save a copy and upload it as part of your submission. DO NOT include this checklist as part of the main manuscript document. It must be uploaded as a separate file.

For peer review only

BMJ Open

A qualitative interview study exploring the barriers and importance of participant diversity in early phase clinical trials

Journal:	*BMJ Open*
Manuscript ID	bmjopen-2023-075547.R1
Article Type:	Original research
Date Submitted by the Author:	15-Dec-2023
Complete List of Authors:	Chatters, Robin; The University of Sheffield, Sheffield Clinical Trials Research Unit (CTRU)<br>Dimairo, Munyaradzi; University of Sheffield, School of Health and Related Research<br>Cooper, Cindy; The University of Sheffield, Sheffield Clinical Trials Research Unit (CTRU)<br>Ditta, Shamila; The University of Sheffield, Sheffield Clinical Trials Research Unit (CTRU)<br>Woodward, Jonathan; The University of Sheffield, Sheffield Clinical Trials Research Unit (CTRU)<br>Biggs, Katie; The University of Sheffield, Sheffield Clinical Trials Research Unit (CTRU)<br>Ogunleye, Della; n/a - patient representative<br>Thistlethwaite, Fiona; The Christie Hospital NHS Trust, The Experimental Cancer Medicine Team; The University of Manchester Division of Cancer Sciences<br>Yap, Christina; Institute of Cancer Research<br>Rothman, Alexander; Sheffield Teaching Hospitals NHS Foundation Trust, Department of Infection, Immunity, and Cardiovascular Disease
**Primary Subject Heading**:	Research methods
Secondary Subject Heading:	Qualitative research
Keywords:	Health Equity, QUALITATIVE RESEARCH, Clinical trials < THERAPEUTICS

SCHOLARONE™
Manuscripts

I, the Submitting Author has the right to grant and does grant on behalf of all authors of the Work (as defined in the below author licence), an exclusive licence and/or a non-exclusive licence for contributions from authors who are: i) UK Crown employees; ii) where BMJ has agreed a CC-BY licence shall apply, and/or iii) in accordance with the terms applicable for US Federal Government officers or employees acting as part of their official duties; on a worldwide, perpetual, irrevocable, royalty-free basis to BMJ Publishing Group Ltd ("BMJ") its licensees and where the relevant Journal is co-owned by BMJ to the co-owners of the Journal, to publish the Work in this journal and any other BMJ products and to exploit all rights, as set out in our licence.

The Submitting Author accepts and understands that any supply made under these terms is made by BMJ to the Submitting Author unless you are acting as an employee on behalf of your employer or a postgraduate student of an affiliated institution which is paying any applicable article publishing charge ("APC") for Open Access articles. Where the Submitting Author wishes to make the Work available on an Open Access basis (and intends to pay the relevant APC), the terms of reuse of such Open Access shall be governed by a Creative Commons licence – details of these licences and which Creative Commons licence will apply to this Work are set out in our licence referred to above.

Other than as permitted in any relevant BMJ Author's Self Archiving Policies, I confirm this Work has not been accepted for publication elsewhere, is not being considered for publication elsewhere and does not duplicate material already published. I confirm all authors consent to publication of this Work and authorise the granting of this licence.

For peer review only

A qualitative interview study exploring the barriers and importance of participant diversity in early phase clinical trials

Robin Chatters (r.chatters@sheffield.ac.uk) , Sheffield Clinical Trials Research Unit, Sheffield Centre for Health and Related Research, The University of Sheffield, Regent Court, Regent Street, S1 4DA (Corresponding author).

Munya Dimairo (m.dimairo@sheffield.ac.uk), Sheffield Clinical Trials Research Unit, Sheffield Centre for Health and Related Research, The University of Sheffield, Regent Street, S1 4DA.

Cindy Cooper (c.l.cooper@sheffield.ac.uk), Sheffield Clinical Trials Research Unit, Sheffield Centre for Health and Related Research, The University of Sheffield, Regent Street, S1 4DA.

Shamila Ditta (s.ditta@sheffield.ac.uk), Sheffield Clinical Trials Research Unit, Sheffield Centre for Health and Related Research, The University of Sheffield, Regent Street, S1 4DA.

Jonathan Woodward (jonathan.woodward@sheffield.ac.uk), Sheffield Clinical Trials Research Unit, Sheffield Centre for Health and Related Research, The University of Sheffield, Regent Street, S1 4DA.

Katie Biggs (c.e.biggs@sheffield.ac.uk), Sheffield Clinical Trials Research Unit, Sheffield Centre for Health and Related Research, The University of Sheffield, Regent Street, S1 4DA.

Della Ogunleye (ogunleyedella5@gmail.com) (patient representative), London, UK.

Fiona Thistlethwaite (fiona.thistlethwaite@nhs.net), The Christie NHS Foundation Trust and University of Manchester, Wilmslow Road, M20 4BX

Christina Yap (christina.yap@icr.ac.uk), Clinical Trials and Statistics Unit, Institute of Cancer Research, Sutton, Cotswold Road, SM2 5NG

Alexander Rothman (a.rothman@sheffield.ac.uk), Department of Infection, Immunity, and Cardiovascular Disease, The Medical School, The University of Sheffield and Sheffield Teaching Hospitals NHS Foundation Trust

Keywords: Equality, diversity, inclusion, early phase trial, qualitative

Word count: 5222

For peer review only

Abstract

Objectives: To explore the importance of, and barriers to achieving, diversity in early phase clinical trials.

Design: In-depth individual qualitative interviews; data analysed using thematic analysis.

Participants: Five professionals (clinical researchers, methodologists with knowledge of early phase clinical trials) and three patient and public representatives (those with experience of early phase clinical trials and/or those from ethnic minority backgrounds).

Results: Professionals viewed that diversity is not currently considered in all early phase clinical trials but felt that it should always be taken into account. Such trials are primarily undertaken at a very small number of centres, thus limiting the populations they can access. Referrals from clinicians based in the community may increase diversity, however, those referred are often not from underserved groups. Referrals may be hindered by the extra resources required to approach and recruit underserved groups and participants often having to undertake "self-driven" referrals. Patient and public representatives stated that diversity is important in research staff too and that potential participants should be informed of the need for diversity in clinical trials. Those from underserved groups may require clarification regarding the potential harms of the treatment, even if these are not known. Education may be key to improving awareness and perception of early phase clinical trials. We provide fourteen recommendations for researchers and funders to improve diversity in early phase clinical trials.

Conclusions: Diversity is important in early phase trials and should be considered in all trials. Consideration is required regarding the extent of diversity and how it is addressed. The increased resources needed to recruit those from underserved groups may warrant funders to increase the funds to support the recruitment of such participants. The potential harms and societal benefits of the research should be presented to potential participants in a balanced but accurate way to increase transparency.

Strengths and limitations of this study

- We qualitatively interviewed a diverse sample of five professionals (clinical researchers and methodologists) and three patient representatives to explore the importance of, and barriers to achieving, diversity in early phase clinical trials.
- Data saturation was not considered; rather, we looked to achieve 'information power', as conceived by Malterud *et al.,* where the size of the study was determined by the amount of information the sample holds.

For peer review only - http://bmjopen.bmj.com/site/about/guidelines.xhtml

- Professional interviewees were representatives from the NHS and UK Universities. All except one interviewee's experience was primarily in UK-based cancer early phase clinical trials. Therefore, the conclusions that can be made regarding other disease areas, sectors and nations are limited.

Background

The importance of equality, diversity and inclusion in clinical research is well documented, with the COVID-19 pandemic bringing into sharp focus the lack of inclusion of underserved groups in clinical trials [1–5]. The value of inclusion of such populations in late phase trials (i.e., in phases 3 and 4) is widely accepted, as treatments may affect diverse subsets of the population differently [6]. As such, there is guidance for researchers to consider groups routinely underserved in research when designing and conducting clinical trials [7–9]. There is evidence to suggest that diversity in such trials may be increased by improving staff competency through training and increased recruitment of staff from diverse backgrounds [6].

There is a lack of guidance regarding the importance of diversity and how to incorporate underserved groups into early phase clinical trials (EPCTs). Two reviews and a Food and Drug Administration (FDA) white paper provide guidance around increasing clinical trial diversity in the drug development process, but all lack guidance specific to EPCTs [10–12]. EPCTs are conducted in healthy volunteers or participants with a specific health condition and help us understand the feasibility of the therapeutic approach, the appropriate dose of the new treatment, how the drug should be administered, how the body responds to the treatment, and side effects. More so, they hugely inform the design and conduct of related later phase trials. Due to their small size and often high-risk nature, EPCTs have specific challenges regarding the inclusion of diverse populations. Despite the unknowns, the current lack of diversity in EPCTs is clear, with research suggesting that EPCTs underrepresent women [13], older adults [14–16], those with lower education levels [17], those who live further away from hospitals [17], those from deprived areas [18], and ethnic minorities [19,20]. It has been shown that participants experiencing socioeconomic deprivation have worse outcomes in cancer trials than those not experiencing socioeconomic deprivation [21], however, we do not know if this is the case for EPCTs due to the lack of data on diversity.

Unfortunately, there is a lack of interventions to address the inequalities within EPCTs [20]. Several studies have been undertaken to assess the barriers to participating in EPCTs, which include the information provided to potential participants [22], expectations of a therapeutic benefit from the trial treatment [23,24], lack of social and financial support, [25] and restrictive eligibility criteria [26].

For peer review only - http://bmjopen.bmj.com/site/about/guidelines.xhtml

However, these studies focus on general recruitment barriers, rather than those that prevent underserved populations from participating. Potential methods of increasing diversity include community outreach, increased diversity of staff, culturally tailored interventions, bilingual study teams [6,27,28], and helping elderly adults navigate barriers to enrolment [29].

Previous research has not discussed the importance of the inclusion of underserved populations in EPCTs, or when it may be important. It may be the case, for certain conditions, treatments, or contexts, that a focus on the representation of the population is less important and therefore diverse populations may not be required. For example, due to suspected homogeneity in how individuals may react to the treatment under investigation. However, excluding certain populations from such trials may lead to an inequitable health policy stacked against underrepresented subpopulations. There is also evidence of a potential 'trial effect' of improved outcomes, greater adherence to clinical guidelines and evidence-based practice of clinicians and institutions that take part in trials [30]. However, the consequences for patient health are uncertain.

We therefore undertook a qualitative study of professionals with knowledge of EPCTs (i.e., clinical researchers and methodologists) and patient and public representatives to explore in which situations diversity may be important in EPCTs, how diversity has been (and could be) incorporated into EPCTs, and the barriers to inclusion in such trials.

Methods

Scope and participant eligibility

We undertook semi-structured qualitative interviews with both professionals (i.e., clinical researchers and methodologists with knowledge of EPCTs) and patient and public representatives (those with experience of EPCTs and/or those from ethnic minority backgrounds). Interviews with professionals explored the level of importance of diversity in EPCTs and when it may be appropriate, their experience of incorporating diversity into EPCTs, and the challenges of doing so. Interviews with the patient and public representatives focussed on the challenges of participating in EPCTs, and methods to improve diversity in such trials.

Recruitment of interview participants

Potential participants were identified by either searching institutional webpages or registries for individuals that are involved in EPCTs (e.g., clinical trials units (CTUs) that are listed on the UK Clinical Research Collaboration (UKCRC) CTU website as having expertise in phase 1 studies); asking study

For peer review only - http://bmjopen.bmj.com/site/about/guidelines.xhtml

collaborators and other contacts for any individuals they are aware of who may be experienced in conducting EPCTs; and via social media (e.g., X, formerly known as Twitter).

Potential participants were sent an email with a participant information sheet (PIS) inviting them to participate in the study. To identify a purposive selection of participants for the interview, individuals were asked to complete a short survey to express their interest in the study which was administered using Qualtrics. The questionnaire requested key demographic data including location, age, ethnicity (patient and public representatives only), experience (professional or patient and public representative), clinical speciality (professionals only), area of expertise (professionals only), and gender. At the end of the survey, respondents were able to indicate whether they were interested in participating in an interview.

Participants were selected purposefully from those that completed the survey (n=22), maximising variation in key characteristics (area of expertise, ethnicity for the patient and public representatives only, years of experience, and gender). Additional participants were approached via email for the interview without having to complete the survey first if they were thought to increase the diversity of the sample.

Research ethics and participant consent process

Consent to participate in the semi-structured interviews was obtained via a consent form, which was completed by the participant prior to the interview, and after completing the survey (if applicable). The participant signed the consent form using an electronic (typed, or image of their signature inserted into the form) signature. The form was then countersigned by the researcher, and a copy of the completed consent form was emailed back to the participant.

Sample size

Due to the potentially large sample size required to achieve data saturation, and the funding and time limitations of the study, data saturation was not considered; rather, we looked to achieve 'information power', as conceived by Malterud *et al.* [31], where the size of the study was determined by the amount of information the sample holds, with studies encompassing narrow aims, dense specificity and strong dialogue having higher information power. We aimed to interview between eight and ten individuals.

Study design and data collection

The study used a phenomenological framework, as interviewees had direct experience of the phenomenon under study. Two semi-structured topic guides (see *supplementary materials*) were used (one for professionals and another for the patient and public representatives). These were

For peer review only - http://bmjopen.bmj.com/site/about/guidelines.xhtml

developed by RC, with the aims of the study used as a starting point. A literature review was undertaken to inform the topic guide, identifying a lack of guidance specifically relating to EPCTs. As a result, literature regarding EDI in later phase trials (III or IV) was used to inform the interview questions [4,6,32]. The topic guides were reviewed by members of the study team, including DO (a patient and public representative), but they were not piloted. The topic guide was iteratively modified after each interview. Seven of the eight interviews were conducted by RC (a male Research Fellow with a BSc), with assistance from JW (a male Research Assistant with an MSc) and SD (a female Research Assistant with an MA). One interview was conducted by SD and JW. All three interviewers had the experience of working on clinical trials within a CTU, had previous experience of qualitative interviewing, and had a general interest in EDI in clinical research. Repeat interviews were not carried out, and transcripts were not returned to the participant for comment or correction. There were no other individuals present at the interviews and interviews lasted from 42 to 62 minutes. Participants undertook the interviewees either whilst at work or home. Field notes were not taken.

All interviews were undertaken via Google Meet, with the audio and video from the interview recorded (with consent) using in-built functionality within the Google Meet platform and transcribed for in-depth analysis. Transcripts were anonymised before analysis.

Emphasis was placed on collecting detailed data from experienced participants. Participants did not provide feedback on the findings. Patient and public interviewees were offered £50 as remuneration for their time.

Relationships of interviewee and interviewers

For most interviewees, there was no prior relationship between the interviewees and the interviewers. However, interviewees would have been aware of the interviewer's interest in this research area. Two interviewees (DO and FT) were involved in the study as collaborators. They provided input into the study protocol and topic guides and were therefore more aware than the other participants of the background, rationale and aims of the study. However, they were not involved in the analysis and only provided feedback on the final written manuscript.

Patient and public involvement

DO (a patient representative with experience of participating in research studies) provided input into all aspects of the study, including the aims and objectives, semi-structured topic guides, and findings.

For peer review only - http://bmjopen.bmj.com/site/about/guidelines.xhtml

Analysis

Data were analysed using thematic analysis, as described by Braun and Clarke [33]. NVivo software (version 12) was used to manage the data. RC and JW read two transcripts in order to familiarise themselves with their contents, then proceeding to independently generate codes through semantic analysis, deriving the themes from the data. Both researchers independently generated themes that related to the research questions and aims. RC and JW met to discuss the themes in order to ensure homogeneity. RC coded the remaining transcripts, adapting the codes where necessary. There were two separate coding trees for the two main participant types – professionals and patient and public representatives, with main themes including the barriers to incorporating diverse populations, the need for diversity in EPCTs, current practice and issues, thoughts on how to improve diversity, and any positive and negative effects of including diverse populations. The two coding trees were combined within the final analysis for readability as they included similar and complementing views on the same topics.

Results

Study flow and characteristics of interviewees

Sixty-eight individuals, based across 31academic, health-care and industry focussed institutions, were approached to participate in the study. Two individuals stated they did not want to take part due to a lack of time and five because of a lack of experience of EPCTs, as well as 53 who did not reply. Five professionals (based within the National Health Service (NHS) and at UK Universities), and three patient and public representatives were interviewed – their characteristics are presented in Table 1. All participants, except one patient and public representative, were based in the UK and had the experience of cancer EPCTs.

Table 1: The characteristics of interviewed participants

For peer review only - http://bmjopen.bmj.com/site/about/guidelines.xhtml

Participant ID	Gender	Ethnicity*	Age range (years)	Role	Years of experience of early phase trials+	Clinical area of experience
Participant 1	Male	Not collected	25- 34	Professional (Operational Lead at a CTU)	6-10	Cancer
Participant 2	Female	Not collected	45-54	Professional (Doctor)	>16	Cancer
Participant 3	Female	Black African	55-64	Patient and public representative	Not collected	Cancer
Participant 4	Female	Not collected	45-54	Professional (Statistician)	11-15	Cancer
Participant 5	Male	Not collected	35-44	Professional (Statistician)	6-10	Cancer
Participant 6	Female	British Chinese	65-74	Patient and public representative	Not collected	Cancer
Participant 7	Female	White Canadian	35-44	Patient and public representative	Not collected	Neurological
Participant 8	Female	Not collected	35-44	Professional (Nurse)	11-15	Cancer

* Ethnicity only collected for the patient and public representatives; CTU, clinical trials unit

+ only collected for professional participants

Views on current practice

Lack of focus on recruiting underserved groups

Professional interviewees stated that they do not currently specifically focus on trying to recruit underserved groups to EPCTs.

> *"I don't think it's something we consciously do. We basically open it to everyone who can enter into the trial, but we do not actively go and look for underserved patients."* (Participant 4, professional)

For peer review only - http://bmjopen.bmj.com/site/about/guidelines.xhtml

Those that did recall conversations on increasing diversity described rudimentary discussions around ensuring variation in the location of sites in the UK (e.g., North or South), and providing translation services, rather than in-depth conversations around how to increase the diversity of their sample.

> *"I think most of the time [discussions around diversity] will be about geography. It will be about "We've got a centre in London and I think I know someone up in Manchester. That'll be good because then we've got somewhere up north."* (Participant 5, professional)

Lack of diversity

It was evident that there is a lack of diversity in EPCTs, with three professionals describing the demographics of those that participate in EPCTs as being generally white, educated, affluent, and young.

> *"We know from our work here that patients coming through to our phase one trials are usually from the largely white, largely middle-class population."* (Participant 2, professional)

Awareness of need for diversity in EPCTs

Participants felt that awareness is increasing, with three interviewees discussing studies they are aware of looking at the level of diversity in EPCTs, and another participant recently becoming aware of a funder requesting information about diversity in grant applications.

> *"The [EPCT] grant application I'm doing at the moment has three questions on [diversity], which we never had before. So, we're having to think about how we are going to address some of these issues."* (Participant 1, professional)

The level of importance for diversity in EPCTs

All interviewees stated that the inclusion of underserved groups is important and should always be considered when undertaking EPCTs.

> *"You'd never want to be ignoring [diversity in EPCTs], I don't think. Trying to think on the spot why you would – I can't think of an argument why you'd not want to consider it."* (Participant 1, professional)

Participants thought that there is a general need to increase diversity in EPCTs without specifically focusing on particular attributes, underserved groups, or by setting a quota for underserved groups in the protocol.

> *"I don't think you can start to tease out who you should and shouldn't exclude in a phase 1 trial, so we should include everyone."* (Participant 2, professional)
>
> *"I don't know if working on a quota system of, say, "We're recruiting 30 participants and we need, in this particular study, we need at least 5 to be black" or "We want a 50:50 split male*

For peer review only - http://bmjopen.bmj.com/site/about/guidelines.xhtml

and female". I don't know how practical that is in the early phase setting." (Participant 5, professional)

There were no situations when ensuring diversity may not be important. Even when undertaking an EPCT in a specific genetic biomarker or disease (e.g., prostate cancer in men), it would still be important to seek diversity in other characteristics (e.g., socioeconomic status).

"[a] prostate cancer study obviously can only recruit males. If you wanted to look at an effect on a certain group or you did want to target a certain socioeconomic group then that could be an area where you'd target and exclude others." (Participant 1, professional)

The need for diversity in EPCTs

Interviewees discussed diversity in EPCTs in terms of the need for exploring treatment signals in diverse populations, research equity, and informing robust design and conduct of later phase trials.

Exploring treatment signals in diverse populations

The most frequently mentioned reason for increasing diversity in EPCTs was to ensure that EPCTs identify side-effects, tolerability, and efficacy in diverse populations early, as different populations may react variably to the study treatment. Many interviewees stated the aim of including diverse populations is to get a "feel" for the toxicities or a "signal" of the efficacy in these populations.

"… in the early phase you're not going to get the statistics to demonstrate that this drug is what the additional toxicities are, or that a different dose should be given or anything like that. I think it's about getting a feel for what the broad categories of toxicities are and what the dose is." (Participant 2, professional)

Improve research equity

A key reason for diversifying clinical trial populations is to improve fairness and to enable underserved groups to access novel treatments.

"It's not fair that there are marginalised groups that are missing out on an opportunity to explore clinical trials and it might be that it's not the right thing for them and that might be the conclusion, but I'd much rather that patient have that informed decision about that before they make that decision." (Participant 8, professional)

Interviewees explained that incorporating diverse groups into EPCTs allows treatments to be identified that may only be efficacious in certain populations. Assuming that treatments that are not efficacious in a Caucasian population do not work for everyone is an injustice if the treatment in fact works in other populations.

For peer review only - http://bmjopen.bmj.com/site/about/guidelines.xhtml

"[If you undertake a] trial [of] a drug in a Caucasian population and it doesn't have any efficacy signals [...] there's an injustice there for the underserved ethnic populations, where actually it could be an efficacious drug." (Participant 2, professional)

Informing robust design and conduct of later phase trials

Improving the design and conduct of future trials was given as a reason to improve diversity, with specific considerations including understanding adherence to the new treatment in other populations, understanding how to better collect trial outcomes across diverse populations, and identifying stratification variables for later phase trials.

Barriers to diversity and methods to improve diversity

Interviewees provided their views on the barriers to incorporating diversity, and associated methods to improve representation, across the following themes: the location of recruiting centres, recruitment practices, the extra resources needed to recruit underserved groups, participant perception (awareness and transparency), and a lack of information on diversity in EPCTs. A summary of the proposed barriers and associated resolutions is provided in Table 2.

Location of recruiting centres in major cities

A main barrier to recruiting diverse populations was seen as the location of centres that undertake EPCTs, which are usually located in the centre of major cities. This can result in only those individuals who can travel to the centre being able to participate in the trial. This is compounded by the intensive time commitment required from participants to participate in these trials (e.g., intensive follow-up schedules).

"I think the main challenge for early phase is actually the fact that it's a lot more intensive if you enter into a trial in the early phase setting, compared to later. If you look at the time schedule of how many times you have to go into clinics, there's a lot more of them in the early phase." (Participant 4, professional)

Two methods were suggested to improve access to the research site. Firstly, both professionals and patient and public representatives suggested that participants and their carers or companions may need to be remunerated for their time (i.e., lost earnings), subsistence, and travel costs.

Secondly, referrals may be made from community hospitals. However, several participants stated that referrals from such centres often do not result in the recruitment of those from underserved groups due to participants often having to initiate the discussion regarding participation in an EPCT. To do so, participants require courage and conviction to ask their doctor about the trial, which may be less likely to be undertaken by those from underserved groups.

"Most of our patients that come to our unit are self-driven referrals [and] are well educated,

For peer review only - http://bmjopen.bmj.com/site/about/guidelines.xhtml

articulate, confident, and would question their consultant and are driven by their own research into clinical trials and what's available and are driving their referral, …But what it does mean is that I feel we are not getting a representative population to even consider a clinical trial." (Participant 8, professional)

Unfortunately, these actions were felt to improve access to the research site, but not the issue of the time commitment required of participants, an issue to which interviewees did not provide a resolution.

"Even if you fund the travel, it's still difficult to have the time [to participate in an EPCT]." (Participant 4, professional)

Unnecessary eligibility criteria

The trial's eligibility criteria may indirectly reduce the diversity of the trial sample, which may be resolved by a patient or diversity representative reviewing the criteria to check for any elements that may inadvertently exclude individuals.

"It might be that you're excluding people who have a certain risk of another particular condition, whether that's a problem with their heart or some other issue … but what you're actually doing is you're making it far more likely that black women would not be eligible for that study because they're more likely to have that particular condition. Prior to the trial we engaged with an EDI representative to think about how we make this project as inclusive as possible. These were the steps we took. Therefore, our inclusion and exclusion criteria now read this, instead of this." (Participant 5, professional)

Participants need to be able to ask questions

Patient and public representatives felt it was important for the participant to be able to ask questions during the recruitment process. However, one professional participant stated many participants often do not feel able to do this, with clinicians also not encouraging questions due to a lack of time. One participant suggested that a sheet of prompts could be given to the participant to refer to during a consultation with their doctor to assist them in asking questions related to the EPCT.

"I developed a question prompt list for patients to have in their hand and they could then refer to the questions that I developed with patients about what they felt they perhaps should have asked at the beginning. it gave the patient permission to ask those questions, [for example] emotive questions like "Will this trial cure my cancer?" (Participant 8, professional)

For peer review only - http://bmjopen.bmj.com/site/about/guidelines.xhtml

Extra resources are needed to recruit underserved groups

Approaching underserved groups to participate in EPCTs was seen as time-consuming. The use of translators to help those without English as their first language to comprehend the trial was seen as a key issue, with this adding to the cost and time demands.

> *"Clinic appointments tend to take longer if there's an interpreter to make sure everything's been explained clearly and that the patient has a good understanding. And ultimately, if a clinic appointment takes longer and you have a high proportion of patients requiring interpreters for example, it potentially reduces the number of patients that you can recruit to a trial."* (Participant 2, professional)

These issues may be intensified in deprived areas due to increased staff turnover and the reliance on locum doctors who lack the time or training to undertake recruitment processes. The increased workload required to recruit those from underserved populations does not result in increased funding.

> *"Someone who's going to bring in patients from more diverse populations isn't going to generate any additional income, because we don't have a supply issue. And actually… [it] might decrease our income because patients who need a translator take longer in clinical time and might be able to treat fewer patients."* (Participant 2, professional)

Therefore, interviewees recommended changes that funders could make, including providing trial teams with extended trial recruitment phases to allow time to recruit those from underserved populations. Additionally, extra per-participant funding for recruiting those from underserved groups may be required.

> *"If you go down to the health economics of it, you've got to have a driver that will benefit us in some way and maybe that's some kind of funding from [the] NIHR, where if a patient is labelled as being from a hard-to-reach population, that patient attracts additional income."* (Participant 2, professional)

Awareness and perception of EPCTs

Lack of awareness of EPCTs

Interviewees suggested that the awareness of EPCTs could be increased. This could be achieved by providing potential participants with access to databases that allow participants to be identified. For example, using a similar portal to the Experimental Cancer Medicine Centres (ECMC) trial finder for cancer EPCTs (which is currently only open to clinicians). Conversations about recruitment to EPCTs should be initiated as early as possible (ideally by the general practitioner, GP). Patient advocacy groups could be used to assist in reaching out to underserved patient groups in a balanced manner.

For peer review only - http://bmjopen.bmj.com/site/about/guidelines.xhtml

"I think it also requires quite a strong patient advocacy group of some sort that currently I don't think exists for EPCTs, which I think you need to reach out for those people to kind of reach out to people who don't normally think about entering into early phase trials. Kind of highlighting pros and cons, being very balanced." (Participant 4, professional)

Perception of EPCTs

Patient and public representatives focussed on their perception of EPCTs. The safety of participating in an EPCT was seen as one of the main barriers to the inclusion of underserved groups, with the perceived risk being high, which was potentially exacerbated by the feeling of being a "guinea pig".

"I think in the Chinese culture, and some others, they are normally equated as guinea pigs and you've got to be self-sacrificing, or prepared to be, before you think about something like this, something drastic might befall you. Which is rather scaremongering." (Participant 6, patient and public representative)

Several professional and patient and public representatives proposed that education may improve awareness and perception of EPCTs, with suggestions including the provision of general health education in schools, educating underserved groups, and educating refers in the need for diversity within EPCTs. However, there was a lack of suggestions on how to achieve this.

*"I think [education is] key. Educating referrers and somehow educating [underserved] groups about the potential for accessing clinical trials. And I don't know fully how that's going to happen, how we can do that effectively." (*Participant 8, professional*)*

Communicating the level of risk associated with participating in an EPCT was seen as key, with safety being a key concern for public representatives, who may want detailed information about what is already known about the drug.

Improving transparency

Transparency was seen as being particularly important. Several methods of increasing transparency were suggested, including providing potential participants with testimonials from other participants and describing the need for diversity to those from underserved groups (i.e., why they are seeking diverse populations to participate in the study, and how they are including such groups).

One participant suggested that researchers should be honest if there is a lack of evidence regarding the safety of a new treatment (e.g., a drug).

"If the researcher feels that "if I share with the potential participant how little actually I know, they will lose confidence." But I would argue that conversely, if you don't share with me what you know and what you don't know, and why am I involved, do I have any valid role

in this? Can I actually make a contribution in some areas? So, in fact, transparency and that openness is confidence enforcing." (Participant 6, patient and public representative)

The need for transparency also extended to what *is not likely* to happen to a participant in an EPCT. One patient and public representative described that underserved populations have a lack of awareness of EPCTs and may assume that there is a significant risk of death. Researchers should therefore clarify the likely adverse events the participant may experience, for example, clarifying that death or paralysis is very unlikely. This could take the form of describing how other people have reacted to the drug.

"Has somebody like me done it before, how did they react to it?... It's just reassuring me that it's gonna be okay, you know, you wouldn't be paralysed." (Participant 3, patient and public representative).

Lack of research staff diversity

A lack of diversity was seen as not only a problem in the research participants themselves, but also in the staff and researchers that undertake and run such studies.

"The more diverse the people conducting the clinical trials, the more diverse it is, the better you see people forthcoming. "Oh, yes, I want to be part of that" When you don't see people like you in it, you know, what effect would it have on me? So, I know we're getting there but, you know, we still need to do more." (Participant 3, patient and public representative)

Lack of information on diversity in EPCTs

Professional participants suggested that little is currently known about the current level of diversity within EPCTs, and therefore, postcode data could be collected for all participants screened for entry into an EPCT to identify if trials are being accessed by underserved groups. However, there may be ethical challenges in collecting such data for those participants who do not consent to participate in the trial.

A schema, depicting the themes and the associations between them, is provided in *Figure 1*.

Discussion

In this qualitative study, we have identified that the diversity of underserved groups does not seem to be currently routinely considered in EPCTs, but it is always viewed as relevant. The main benefit of including diverse populations in EPCTs is the identification of early signals of harms and efficacy in diverse populations, including underserved subpopulations, and providing equitable opportunities for patients. Study teams should make a concerted effort to recruit those from underserved groups into their EPCT and should seek to minimise the numerous barriers that prevent such individuals from participating. The most notable barrier appeared to be that EPCTs are primarily undertaken at

For peer review only - http://bmjopen.bmj.com/site/about/guidelines.xhtml

a small number of specialist centres, predominately located within the centre of large cities, thus limiting the populations they can access. To increase diversity, these centres may need to rely on referrals from "feeder" centres located in the community. However, the extra resources it requires to approach and recruit underserved populations (e.g., language translation) can prevent clinicians from referring them. Patient and public representatives stated that trust and communication are important – those recruiting underserved groups to EPCTs should be transparent regarding the need for diversity in clinical trials, the potential harms of the treatment, and if these are not known, clarifying the likelihood of severe illness to ease their concerns. Education of both patients/public and clinical staff was felt to be key to increasing awareness and perception of EPCTs.

The strengths of this study are that both professionals and patient and public representatives were sought to obtain the views of two of the key stakeholders involved in EPCTs. Both sets of individuals were diverse, in terms of the role of professional participants (including clinicians, statisticians, and operational leads) and the ethnicity of the patient and public representatives. Due to funding limitations only a small number of interviews were undertaken. Additionally, the approvals necessary to interview staff in the National Health Service (NHS) were not sought due to time limitations, and therefore only those staff that also had contracts with a University were interviewed, thus limiting the sample to those NHS staff that also had academic posts. Those from other organisations (e.g., the pharmaceutical industry) were not interviewed. Additionally, all except one interviewee's experience was primarily in UK-based cancer EPCTs, therefore limiting the conclusions that can be made regarding other disease areas and nations. Unfortunately, funding was not available to undertake additional recruitment to interview those with expertise outside of cancer trials research. Lastly, there was no provision for those individuals who required assistance to converse in English, which may have prohibited those with significant barriers to taking part in EPCTs from participating in this study.

This study supports the literature regarding the need to increase diversity in clinical trials [6,34]. Some of the barriers and methods to increase diversity have been reported previously across both early and late phase trials, including adequately funding participants' time and travel costs [35], restrictive eligibility criteria [12], burdensome trial procedures [12], informing community oncologists of the trial to aid recruitment [29], and translation and language issues [27]. We have identified that, due to time constraints, those who refer to clinical trials may not approach those from underserved groups, which has been found in a previous study [36]. Our study adds to this literature by clarifying that diversity is important in all EPCTs, and by identifying additional barriers that, as far as we are aware, have not been previously reported. These additional barriers concern

For peer review only - http://bmjopen.bmj.com/site/about/guidelines.xhtml

the effect of the location of EPCT units on their ability to access underserved groups and the lack of referrals to these units of underserved groups from referrers in the community. Such referrals may be hindered by the need for participants to “self-drive” the referral, which may be a barrier given the lack of knowledge of EPCTs in underserved groups. We have also identified specific issues related to transparency, including informing the participant the reason for the need to focus on the recruitment of underserved groups. Interviewees also suggested that an indication of the level of risk associated with the EPCT is provided during the recruitment process, even if the effects of the drug are unknown. It is important to note that, as per Good Clinical Practice (GCP) guidelines, the risks of participating in research should always be communicated to patients [37]. However, participants in this study desired an estimation of the risks of research even when these cannot be quantified, for example, reassurance that the drug under test is unlikely to lead to death or significant injury.

This study implies that those designing and recruiting to EPCTs should attempt to increase the diversity of their sample by referring to the fourteen principles outlined in *Table 3*. In this table, the authors have supplemented the findings from the qualitative study with their own reflections, which are provided in *italics*.

It should be noted that given that those participating in EPCTs may have optimistic expectations of the effect of participating in an EPCT [24], highlighting the harms of such trials may have a negative effect on recruitment, so researchers may also describe to participants the potential societal benefits of EPCTs highlighted in this study (e.g., improving the equity of research, exploring early treatment signals in diverse populations, and informing the robust design of future trials). Future research should focus on exploring the barriers and enablers to recruitment in a larger diverse sample of participants, including NHS staff, and by investigating how potential harms are communicated to participants, especially in the absence of quantifiable data. Overcoming the inherent barriers to undertaking clinical trials in novel treatments, which, due to their risk, often require participants having to regularly attend the hospital for safety checks, may be challenging but would open up EPCTs to patient groups who are currently unable to participate in such trials. These “remote” EPCTs have been broached, but are rarely undertaken in practice [38]. Additionally, educational packages could be developed to increase awareness and improve the perception of EPCTs in underserved groups – future research may focus on the content, audience, timing, and format of such packages.

For peer review only - http://bmjopen.bmj.com/site/about/guidelines.xhtml

Declarations

Author contributorship

RC, MD and CC devised the study. RC, SD and JW collected the data and undertook the analysis. RC wrote the first draft of the report. DO was a patient representative who provided input into the design of the study and the findings. All authors, including AR, CY, FT and KB, provided input into the design and implementation of the study, critically reviewed the draft, and provided approval for publication.

Competing interests

The authors declare that they have no competing interests.

Funding

The study was funded by Research England via the Participatory Research Fund (X/175529).

Data sharing

The dataset is the in-depth interview transcripts. Access is restricted to the study team in accordance with the requirements of the ethical approval.

Patient consent

Participants provided informed consent to participate in the study and for their quotations to be anonymously published.

Ethical approval

The study received favourable ethics approval from the research ethics committee based at the School of Health and Related Research (ScHARR), within the University of Sheffield (reference number: 045712).

Acknowledgements

We would like to thank the eight participants for their time in participating in the interviews and for sharing their valuable knowledge with us.

In memory of Dolapo (Della) Ogunleye, whose passion, insights and dedication to improving diversity and inclusion of participants in research to achieve equitable patient care, enriched every aspect of this work

References

1 Diversity Data Report. Published Online First: 2021.https://www.nihr.ac.uk/about-us/our-key-priorities/equality-diversity-and-inclusion/diversity-data-report.htm

For peer review only - http://bmjopen.bmj.com/site/about/guidelines.xhtml

For peer review only

2 UK government sets out bold vision for the future of clinical research delivery - GOV.UK. https://www.gov.uk/government/news/uk-government-sets-out-bold-vision-for-the-future-of-clinical-research-delivery (accessed 12 Jan 2022).

3 Inclusion of Women and Minorities as Participants in Research Involving Human Subjects | grants.nih.gov. https://grants.nih.gov/policy/inclusion/women-and-minorities.htm (accessed 12 Jan 2022).

4 Improving inclusion of under-served groups in clinical research: Guidance from INCLUDE project. https://www.nihr.ac.uk/documents/improving-inclusion-of-under-served-groups-in-clinical-research-guidance-from-include-project/25435 (accessed 12 Jan 2022).

5 UKRI equality diversity and inclusion strategy: draft for consultation – UKRI. https://www.ukri.org/publications/equality-diversity-and-inclusion-strategy-draft-for-consultation/ukri-equality-diversity-and-inclusion-strategy-draft-for-consultation/ (accessed 17 Jan 2022).

6 Bodicoat DH, Routen AC, Willis A, *et al.* Promoting inclusion in clinical trials—a rapid review of the literature and recommendations for action. *Trials* 2021;**22**. doi:10.1186/S13063-021-05849-7

7 Treweek S, Banister K, Bower P, *et al.* Developing the INCLUDE Ethnicity Framework—a tool to help trialists design trials that better reflect the communities they serve. *Trials* 2021;**22**:1–12. doi:10.1186/S13063-021-05276-8/TABLES/3

8 Jutlla K. INCREASING PARTICIPATION OF BLACK ASIAN AND MINORITY ETHNIC (BAME) GROUPS IN HEALTH AND SOCIAL CARE RESEARCH. 2018.

9 Equality Impact Assessment Guidance and Template. http://www.acas.org.uk/media/pdf/s/n/Acas_managers_guide_to_equality_assessments.pdf (accessed 31 Aug 2022).

10 Moore KT, Grundmann O, Iwuchukwu O, *et al.* The Importance of Diversity and Inclusion in Drug Development and Clinical Trial Conduct. *J Clin Pharmacol* 2022;**62**:1475–9. doi:10.1002/jcph.2151

11 Gross AS, Harry AC, Clifton CS, *et al.* Clinical trial diversity: An opportunity for improved insight into the determinants of variability in drug response. *Br J Clin Pharmacol* 2022;**88**:2700–17. doi:10.1111/bcp.15242

For peer review only - http://bmjopen.bmj.com/site/about/guidelines.xhtml

12 Enhancing the Diversity of Clinical Trial Populations — Eligibility Criteria, Enrollment Practices, and Trial Designs Guidance for Industry. 2020. https://www.fda.gov/media/127712/download

13 Pinnow E, Sharma P, Parekh A, *et al.* Increasing Participation of Women in Early Phase Clinical Trials Approved by the FDA. *Women's Heal Issues* 2009;**19**:89–93. doi:10.1016/j.whi.2008.09.009

14 Baldini C, Charton E, Schultz E, *et al.* Access to early-phase clinical trials in older patients with cancer in France: the EGALICAN-2 study. *ESMO Open* 2022;**7**:100468. doi:10.1016/J.ESMOOP.2022.100468

15 Khan KH, Yap TA, Ring A, *et al.* Phase I trial outcomes in older patients with advanced solid tumours. *Br J Cancer* 2016;**114**:262–8. doi:10.1038/bjc.2015.477

16 Mahipal A, Denson AC, Djulbegovic B, *et al.* Effect of Age on Clinical Outcomes in Phase 1 Trial Participants. *Cancer Control* 2015;**22**:235–41. doi:10.1177/107327481502200217

17 Gad KT, Johansen C, Duun-Henriksen AK, *et al.* Socioeconomic differences in referral to phase i cancer clinical trials: A danish matched cancer case-control study. *J Clin Oncol* 2019;**37**:1111–9. doi:10.1200/JCO.18.01983

18 Mohd Noor A, Sarker D, Vizor S, *et al.* Effect of Patient Socioeconomic Status on Access to Early-Phase Cancer Trials. *J Clin Oncol* 2013;**31**:224–30. doi:10.1200/JCO.2012.45.0999

19 Dunlop HM, Fitzpatrick E, Kurti K, *et al.* Abstract PR-05: Worsening disparities of racial minority participation in phase 1 early drug development trials in the United States, 2001-2018. *Cancer Epidemiol Biomarkers Prev* 2022;**31**:PR-05-PR-05. doi:10.1158/1538-7755.DISP21-PR-05

20 Perni S, Moy B, Nipp RD. Disparities in phase 1 cancer clinical trial enrollment. *Cancer* 2021;**127**:4464–9. doi:10.1002/cncr.33853

21 Unger JM, Moseley AB, Cheung CK, *et al.* Persistent Disparity: Socioeconomic Deprivation and Cancer Outcomes in Patients Treated in Clinical Trials. *J Clin Oncol* 2021;**39**:1339–48. doi:10.1200/JCO.20.02602

22 Cox K. Informed consent and decision-making: patients' experiences of the process of recruitment to phases I and II anti-cancer drug trials. *Patient Educ Couns* 2002;**46**:31–8. doi:10.1016/s0738-3991(01)00147-1

For peer review only - http://bmjopen.bmj.com/site/about/guidelines.xhtml

23 Meropol NJ, Weinfurt KP, Burnett CB, *et al.* Perceptions of patients and physicians regarding phase I cancer clinical trials: implications for physician-patient communication. *J Clin Oncol* 2003;**21**:2589–96. doi:10.1200/JCO.2003.10.072

24 Catt S, Langridge C, Fallowfield L, *et al.* Reasons given by patients for participating, or not, in Phase 1 cancer trials. *Eur J Cancer* 2011;**47**:1490–7. doi:10.1016/j.ejca.2011.02.020

25 Castillo G, Lalu MM, Asad S, *et al.* Navigating choice in the face of uncertainty: using a theory informed qualitative approach to identifying potential patient barriers and enablers to participating in an early phase chimeric antigen receptor T (CAR-T) cell therapy trial. *BMJ Open* 2021;**11**:e043929. doi:10.1136/bmjopen-2020-043929

26 Ho J, Pond GR, Newman C, *et al.* Barriers in phase I cancer clinical trials referrals and enrollment: five-year experience at the Princess Margaret Hospital. *BMC Cancer* 2006;**6**:263. doi:10.1186/1471-2407-6-263

27 Chalela P. Promoting Factors and Barriers to Participation in Early Phase Clinical Trials: Patients Perspectives. *J Community Med Health Educ* 2014;**04**. doi:10.4172/2161-0711.1000281

28 Pham B, Earle N, Rabel K, *et al.* Maximizing the diversity of participants in a phase II clinical trial of optical technologies to detect cervical neoplasia. *Gynecol Oncol* 2007;**107**:S208–14. doi:10.1016/J.YGYNO.2007.07.063

29 Basche M, Barón AE, Eckhardt SG, *et al.* Barriers to Enrollment of Elderly Adults in Early-Phase Cancer Clinical Trials. *J Oncol Pract* 2008;**4**:162–8. doi:10.1200/JOP.0842001

30 Clarke M, Loudon K. Effects on patients of their healthcare practitioner's or institution's participation in clinical trials: a systematic review. *Trials* 2011;**12**:16. doi:10.1186/1745-6215-12-16

31 Malterud K, Siersma VD, Guassora AD. Sample Size in Qualitative Interview Studies. *Qual Health Res* 2016;**26**:1753–60. doi:10.1177/1049732315617444

32 Ford JG, Howerton MW, Lai GY, *et al.* Barriers to recruiting underrepresented populations to cancer clinical trials: A systematic review. *Cancer* 2008;**112**:228–42. doi:10.1002/CNCR.23157

33 Braun, V., Clark V. Using thematic analysis in psychology. *Qual Res Psychol* 2006;**3**:77–101.

34 Inclusion of Older Adults in Cancer Clinical Trials Guidance for Industry.

For peer review only - http://bmjopen.bmj.com/site/about/guidelines.xhtml

https://www.fda.gov/media/156616/download#:~:text=The issue of older adults being underrepresented in,malignancy%2C all of which may increase with age.

35 Bierer BE, White SA, Gelinas L, *et al.* Fair payment and just benefits to enhance diversity in clinical research. *J Clin Transl Sci* 2021;**5**:e159. doi:10.1017/cts.2021.816

36 Gomes F, Descamps T, Lowe J, *et al.* Enrolment of older adults with cancer in early phase clinical trials—an observational study on the experience in the north west of England. *Age Ageing* 2021;**50**:1736–43. doi:10.1093/ageing/afab091

37 Guideline for good medical practice E6(R2). https://www.ema.europa.eu/en/documents/scientific-guideline/ich-guideline-good-clinical-practice-e6r2-step-5_en.pdf

38 Alemayehu D, Hemmings R, Natarajan K, *et al.* Perspectives on Virtual (Remote) Clinical Trials as the "New Normal" to Accelerate Drug Development. *Clin Pharmacol Ther* 2022;**111**:373–81. doi:10.1002/cpt.2248

Table 2: Barriers and facilitators to increasing diversity in EPCTs

**Category**	**Barrier**	**Facilitator**
Patient factors	EPCTs require intensive time commitment from trial participants, which may deter involvement	None discussed
	Participants may have to undertake "self-driven" referrals and require the motivation and courage to initiate discussions regarding EPCTs with their clinician	• Educate potential participants regarding the EPCTs that are available to them<br>• Educate clinicians to enable them to approach and recruit participants to EPCTs
	A perceived lack of transparency may result in those from underserved groups being unlikely to participate in an EPCT	• Provide potential participants with testimonials from other participants<br>• Describe the need for diversity in the EPCT to those from underserved

For peer review only - http://bmjopen.bmj.com/site/about/guidelines.xhtml

		groups and the importance of participating
	It is important for participants to ask questions about the EPCT when discussing their potential participation, but participants may not ask questions, and clinicians may not encourage them, due to a lack of time	• A list of potential questions can be given to participants to prompt them during a consultation with their doctor/clinician
	There is a lack of awareness of EPCTs amongst underserved groups	• Discussions around participating in an EPCT should be initiated as early as possible, ideally by the GP<br>• Education to improve awareness, both for patients and clinical staff.<br>• Permit use of trial databases to allow participants to locate EPCTs (e.g., Experimental Cancer Medicine Centres (ECMC) in cancer).<br>• Patient advocacy groups could be established to assist in relaying information to underserved groups in a balanced manner.
	Those from underserved groups may have concerns around the safety of participating in an EPCT	• Communicate the level of risk associated with the EPCT with the participant<br>• Provide detailed information about what is already known about study treatment<br>• Provide detail as to what is unlikely to happen to participants (e.g., death, paralysis)<br>• Educate patients to help improve their perception of EPCTs.

For peer review only - http://bmjopen.bmj.com/site/about/guidelines.xhtml

Patient factors / trial design and implementation	Many centres that recruit to EPCTs are located in the centre of major cities which may be challenging for certain populations to access	• Referrals can be made from community hospitals<br>• Provide reimbursement to trial participants and their carers for travel and subsistence costs
Trial design and implementation	A lack of diversity in trial staff and researchers may deter participants from taking part	• Increase the diversity of staff that undertake and run EPCTs
	Eligibility criteria may inadvertently reduce the diversity of the trial sample	• Engage with a patient or diversity representative to review eligibility criteria
	Approaching underserved groups to participate in an EPCT requires extra resources	• Additional funding and time may be required to recruit those from underserved groups

Table 3: Recommendations for researchers and funders

**Recommendations for researchers**
1) Increasing the diversity of the sample should always be considered in EPCTs to a) explore treatment signals in diverse populations, b) improve research equity, and c) inform the robust design and conduct of later phase trials. *Some diseases have specific diversity profiles, which should be reflected in the trial sample.*
2) Those undertaking EPCTs should make a concerted effort to recruit those from underserved groups. The diversity of EPCTs should generally be increased, rather than focussing on specific attributes or quotas.
3) Important markers of diversity (e.g. postcode, *ethnicity, age*) should be consistently collected and report from all those that are screened to participate in EPCTs.
4) In order to reach diverse communities, potential participants may be referred from community hospitals. However, participants may still struggle to access the main trial site, so reimbursement for participants and their carers time and travel costs should

For peer review only - http://bmjopen.bmj.com/site/about/guidelines.xhtml

be considered. *Enhancing participant awareness of the reimbursement of travel and other costs is important.*
5) Education of both participants and staff is important to ensure both parties are aware of EPCTs, in order to enable recruitment and retention.
6) Researchers should ensure eligibility criteria do not inadvertently exclude those from underserved groups and should seek input from patient or diversity representatives into these criteria. *However, this should be balanced with the risk of the EPCT – some exclusions may be justified based on safety concerns.*
7) A list of prompts may be provided to participants to refer to during conversations regarding participation in an EPCT to assist them in asking questions about the study.
8) Discussions around entry into an EPCT should commence as early as possible in the participant's journey, ideally with the GP.
9) Participants from underserved groups may be concerned about the risks of participating in an EPCT. In order to reassure them, researchers may:<br>a. Clearly communicate the level of risk associated with the EPCT with the participant. The societal benefits of participating in an EPCT may also be relayed to participants (see principle 1);<br>b. Provide detailed information about what is already known about the study treatment;<br>c. Provide an honest explanation of the amount available regarding the evidence regarding the effects of the study treatment;<br>d. Provide information as to what is *unlikely* to happen to participants as a result of the study treatment (e.g., death, paralysis).
10) In order to assist in relaying information to underserved groups in a balanced manner, patient advocacy groups may be formed.
11) Due to a perceived lack of transparency, those from underserved groups may be provided with testimonials from other participants. The need for diversity in the EPCT may be explained to such individuals.
12) The diversity of those staff that recruit to EPCTs should be increased in order to not deter underserved groups from participating.

For peer review only - http://bmjopen.bmj.com/site/about/guidelines.xhtml

**Recommendations for funders**
13) Funders may need to provide additional per participant funds for the recruitment of those from underserved groups due to the additional resources required to recruit such individuals.
14) Funders should request details of the methods investigators will use to identify, approach and recruit those from underserved groups in grant applications.

For peer review only

For peer review only - http://bmjopen.bmj.com/site/about/guidelines.xhtml

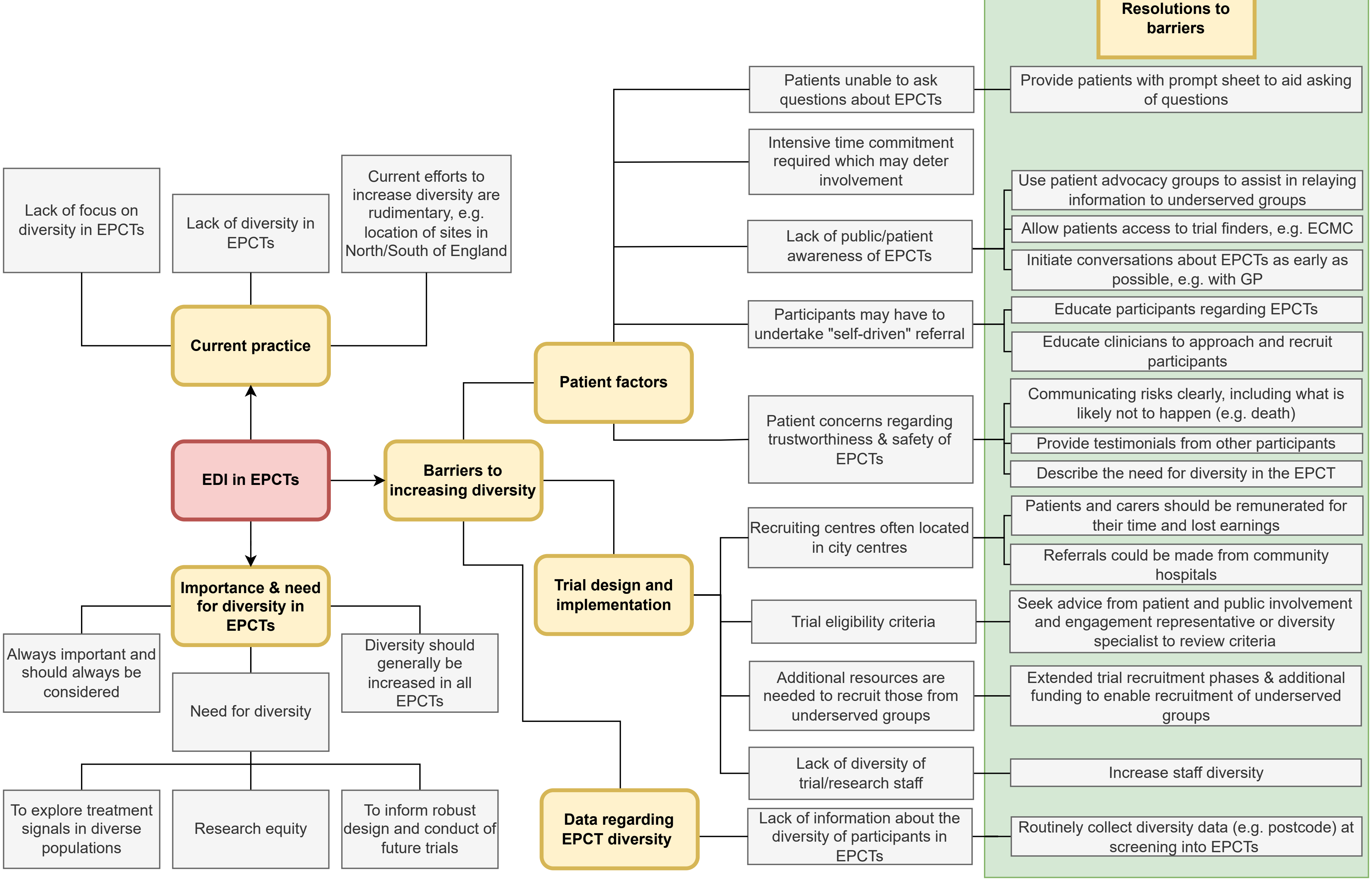

Figure 1: Thematic Schema

EDI in early phase trials – qualitative interview topic guide (patients and research participants)

NOTES: This topic guide is a flexible tool and may be revised as new areas of interest arise during the process of data collection. The wording of questions is for guidance only and can be varied to suit the natural style of the interviewer and the level of understanding of the participant.

1. Welcome and context setting:

- Introduce yourself
- Guide the participant through the Participant Information Sheet
- "The interview will last approximately 45 to 60 minutes"
- Explain that, "to help us with this study, we would like to make a recording of what we all say today, but nobody will be able to identify you from that recording other than me. Is that okay?"
- Guide the participant through the consent form
- "You are free to withdraw at any point during the interview and you don't have to answer any of the questions if you don't want to, but once the interview has finished I will be unable to remove your information from the study, if you chose to withdraw"
- Check they are happy to continue and ask if there are any questions.

2. Describe to the interviewee the definitions of underserved groups and EDI (in lay person language) we are using (these will also be provided to participants to refer to):

- Remind participant why we are doing this research
- Give brief definition of "EDI"
- Give brief definition of an early phase trial

3. Questions

QUESTION 1: What do you understand of early phase trials?

PROMPT: Have you been approached to take part in such a study?

QUESTION 2: What is your experience of the process of being approached to take part, or taking part in, early phase trials?

PROMPT: How were you approached to take part?
PROMPT: How did you find being approached?
PROMPT: How did you make the decision as to whether or not to take part?
PROMPT: What factors influenced your decision to either participate, or not participate?

QUESTION 3: What is your experience of taking part (or being approached to take part in) early phase trials?

PROMPT: What challenges were there to you participating in early phase trials?

For peer review only - http://bmjopen.bmj.com/site/about/guidelines.xhtml

Cultural and institutional barriers, attitudes and beliefs, emotional and psychological barriers, financial barriers

PROMPT: Were there any enablers?

PROMPT: What challenges are there for others who are from underserved groups?

PROMPT: anything else you would like to mention?

QUESTION 4: What influences your decision regarding whether or not to participate in early phase trials?

QUESTION 5: What enables you to take part in early phase trials?

PROMPT: What challenges are there to you participating in early phase trials?

PROMPT: What challenges are there for others who are from underserved groups?

QUESTION 6: What benefits are there to taking part in these trials?

PROMPT: What benefits are there to you participating in early phase trials?

PROMPT: What benefits are there for others who are from underserved groups?

QUESTION 7: What can be done to encourage more people to participate in early phase trials?

For peer review only - http://bmjopen.bmj.com/site/about/guidelines.xhtml

EDI in early phase trials – qualitative interview topic guide

NOTES: This topic guide is a flexible tool and may be revised as new areas of interest arise during the process of data collection. The wording of questions is for guidance only and can be varied to suit the natural style of the interviewer and the level of understanding of the participant.

1. Welcome and context setting:

- Introduce yourself
- Remind the participant of the aims of the study
- Guide the participant through the Participant Information Sheet
- "The interview will last approximately 45 to 60 minutes"
- Explain that, "to help us with this study, we would like to make a recording of what we all say today, but nobody will be able to identify you from that recording other than me. Is that okay?"
- Guide the participant through the consent form
- "You are free to withdraw at any point during the interview and you don't have to answer any of the questions if you don't want to, but once the interview has finished I will be unable to remove your information from the study, if you chose to withdraw"
- Check they are happy to continue and ask if there are any questions.

Remind the participants of the definition of underserved groups:

We are using the National Institute for Health Research's (NIHR's) guidance from the INnovations in CLinical trial design and delivery for the UnDEr-served (INCLUDE) project to define an underserved group, which states that:

> "The work of the NIHR-INCLUDE project shows that there is no single definition for an under-served group. Some key characteristics that are common to several under-served groups are:
>
> - Lower inclusion in research than one would expect from population estimates
> - High healthcare burden that is not matched by the volume of research designed for the group
> - Important differences in how a group responds to or engages with healthcare interventions compared to other groups, with research neglecting to address these factors
>
> "A key finding from our work is that the definition of 'under-served' is often very context and study specific. An under-served group for one disease or type of study may be the opposite to that of another."

EDI may be important due to:

- Lower inclusion of underserved groups in research than one would expect from population estimates
- High healthcare burden that is not matched by the volume of research designed for the group

For peer review only - http://bmjopen.bmj.com/site/about/guidelines.xhtml

- Important differences in how a group responds to or engages with healthcare interventions compared to other groups, with research neglecting to address these factors

2. Questions (remind participant to give examples, where appropriate)

QUESTION 1: Generally, what do you think is the value of including underserved groups in early phase trials?

PROMPT: Is there any value to including underserved groups in early phase trials?

PROMPT: Should early phase trials provide equal access to underserved groups (diversity)?

PROMPT: Should early phase trials recognise differences between individuals?

PROMPT: What are the positive consequences?

PROMPT: What are the negative consequences?

PROMPT: Is the value of underserved groups in early phase different from late phase and if so how?

QUESTION 2: When may the inclusion of underserved groups in early phase trials be warranted, and unwarranted (with reasons)?

PROMPT: Are there any situations (e.g. diseases, treatments) where underserved groups is required? If so, why?

PROMPT: Are there any situations (e.g. diseases, treatments) where underserved groups is less important? If so, why?

QUESTION 3: What is your view on the outcome of including underserved groups in early phase trials?

PROMPT: Does including (or not including) underserved groups affect future, later phase trials?

PROMPT: Does including (or not including) underserved groups affect the regulatory process?

QUESTION 4: Have you incorporated underserved groups into any early phase trials you have conducted?

PROMPT: Did you specifically plan any strategies to include individuals from underserved groups? Which groups?

PROMPT: Why did you (or why didn't you) look to include underserved groups in the trial?

QUESTION 5: How did you (OR HOW WOULD YOU) incorporate underserved groups into previous early phase trials?

PROMPT: How did you identify and approach those from underserved groups?

PROMPT: Were there any other design considerations taken into account? E.g. translation, reducing burden, amending exclusion criteria.

For peer review only - http://bmjopen.bmj.com/site/about/guidelines.xhtml

QUESTION 6 What are the challenges of including underserved groups in early phase trials? How have you, or would you, overcome these?

PROMPT: Have you encountered any challenges when incorporating underserved groups?

PROMPT: How did you, or might you overcome these challenges?

(if not already mentioned): Are there any challenges with regards to incorporating multiple underserved groups in a small early phase trial?

QUESTION 7: What enables (or helps) the incorporation of underserved groups into early phase trials?

Describe the definitions of underserved groups and EDI we are using (these will also be provided to participants to refer to). Ask participant to reflect on these definitions:

EDI:

Equality – Individuals are not treated less favourably due to their characteristics and have equal opportunities to take part in early phase trials, and are provided with equal opportunities if they decide to take part in a trial.

QUESTION: What are your reflections on this definition? Would you change it in any way?

QUESTION: What does equality mean in the context of early phase trials?

QUESTION: Do you think early phase trials provide equal access to groups (and do you think they should)?

QUESTION: Do you think people from particular groups are excluded from early phase trials?

Diversity – Recognising and respecting differences in individuals who are approached to take part, and decide to take part, in early phase trials.

QUESTION: What are your reflections on this definition? Would you change it in any way?

QUESTION: What does diversity mean in the context of early phase trials?

QUESTION: Do you think early phase trials recognise and respect the differences of individuals? Do you think they should?

Inclusion – Creating an environment where everyone feels welcomed and valued.

QUESTION: What does inclusion mean in the context of early phase trials?

QUESTION: Do you think early phase trials are inclusive? Do you think they should be?

For peer review only - http://bmjopen.bmj.com/site/about/guidelines.xhtml

COREQ (COnsolidated criteria for REporting Qualitative research) Checklist

A checklist of items that should be included in reports of qualitative research. You must report the page number in your manuscript where you consider each of the items listed in this checklist. If you have not included this information, either revise your manuscript accordingly before submitting or note N/A.

Topic	Item No.	Guide Questions/Description	Reported on Page No.
**Domain 1: Research team and reflexivity**			
Personal characteristics			
Interviewer/facilitator	1	Which author/s conducted the interview or focus group?	
Credentials	2	What were the researcher's credentials? E.g. PhD, MD	
Occupation	3	What was their occupation at the time of the study?	
Gender	4	Was the researcher male or female?	
Experience and training	5	What experience or training did the researcher have?	
Relationship with participants			
Relationship established	6	Was a relationship established prior to study commencement?	
Participant knowledge of the interviewer	7	What did the participants know about the researcher? e.g. personal goals, reasons for doing the research	
Interviewer characteristics	8	What characteristics were reported about the inter viewer/facilitator? e.g. Bias, assumptions, reasons and interests in the research topic	
**Domain 2: Study design**			
Theoretical framework			
Methodological orientation and Theory	9	What methodological orientation was stated to underpin the study? e.g. grounded theory, discourse analysis, ethnography, phenomenology, content analysis	
Participant selection			
Sampling	10	How were participants selected? e.g. purposive, convenience, consecutive, snowball	
Method of approach	11	How were participants approached? e.g. face-to-face, telephone, mail, email	
Sample size	12	How many participants were in the study?	
Non-participation	13	How many people refused to participate or dropped out? Reasons?	
Setting			
Setting of data collection	14	Where was the data collected? e.g. home, clinic, workplace	
Presence of non-participants	15	Was anyone else present besides the participants and researchers?	
Description of sample	16	What are the important characteristics of the sample? e.g. demographic data, date	
Data collection			
Interview guide	17	Were questions, prompts, guides provided by the authors? Was it pilot tested?	
Repeat interviews	18	Were repeat inter views carried out? If yes, how many?	
Audio/visual recording	19	Did the research use audio or visual recording to collect the data?	
Field notes	20	Were field notes made during and/or after the inter view or focus group?	
Duration	21	What was the duration of the inter views or focus group?	
Data saturation	22	Was data saturation discussed?	
Transcripts returned	23	Were transcripts returned to participants for comment and/or	

For peer review only

Topic	Item No.	Guide Questions/Description	Reported on Page No.
		correction?	
**Domain 3: analysis and findings**			
Data analysis			
Number of data coders	24	How many data coders coded the data?	
Description of the coding tree	25	Did authors provide a description of the coding tree?	
Derivation of themes	26	Were themes identified in advance or derived from the data?	
Software	27	What software, if applicable, was used to manage the data?	
Participant checking	28	Did participants provide feedback on the findings?	
Reporting			
Quotations presented	29	Were participant quotations presented to illustrate the themes/findings? Was each quotation identified? e.g. participant number	
Data and findings consistent	30	Was there consistency between the data presented and the findings?	
Clarity of major themes	31	Were major themes clearly presented in the findings?	
Clarity of minor themes	32	Is there a description of diverse cases or discussion of minor themes?	

Developed from: Tong A, Sainsbury P, Craig J. Consolidated criteria for reporting qualitative research (COREQ): a 32-item checklist for interviews and focus groups. *International Journal for Quality in Health Care*. 2007. Volume 19, Number 6: pp. 349 – 357

Once you have completed this checklist, please save a copy and upload it as part of your submission. DO NOT include this checklist as part of the main manuscript document. It must be uploaded as a separate file.

For peer review only

Exploring the barriers to, and importance of, participant diversity in early phase clinical trials: an interview-based qualitative study of professionals and patient and public representatives

Journal:	*BMJ Open*
Manuscript ID	bmjopen-2023-075547.R2
Article Type:	Original research
Date Submitted by the Author:	08-Feb-2024

Complete List of Authors:	Chatters, Robin; The University of Sheffield, Sheffield Clinical Trials Research Unit (CTRU)<br>Dimairo, Munyaradzi; University of Sheffield, School of Health and Related Research<br>Cooper, Cindy; The University of Sheffield, Sheffield Clinical Trials Research Unit (CTRU)<br>Ditta, Shamila; The University of Sheffield, Sheffield Clinical Trials Research Unit (CTRU)<br>Woodward, Jonathan; The University of Sheffield, Sheffield Clinical Trials Research Unit (CTRU)<br>Biggs, Katie; The University of Sheffield, Sheffield Clinical Trials Research Unit (CTRU)<br>Ogunleye, Della; n/a - patient representative<br>Thistlethwaite, Fiona; The Christie Hospital NHS Trust, The Experimental Cancer Medicine Team; The University of Manchester Division of Cancer Sciences<br>Yap, Christina; Institute of Cancer Research<br>Rothman, Alexander; Sheffield Teaching Hospitals NHS Foundation Trust, Department of Infection, Immunity, and Cardiovascular Disease

**Primary Subject Heading**:	Research methods
Secondary Subject Heading:	Qualitative research
Keywords:	Health Equity, QUALITATIVE RESEARCH, Clinical trials < THERAPEUTICS

SCHOLARONE™ Manuscripts

I, the Submitting Author has the right to grant and does grant on behalf of all authors of the Work (as defined in the below author licence), an exclusive licence and/or a non-exclusive licence for contributions from authors who are: i) UK Crown employees; ii) where BMJ has agreed a CC-BY licence shall apply, and/or iii) in accordance with the terms applicable for US Federal Government officers or employees acting as part of their official duties; on a worldwide, perpetual, irrevocable, royalty-free basis to BMJ Publishing Group Ltd ("BMJ") its licensees and where the relevant Journal is co-owned by BMJ to the co-owners of the Journal, to publish the Work in this journal and any other BMJ products and to exploit all rights, as set out in our licence.

The Submitting Author accepts and understands that any supply made under these terms is made by BMJ to the Submitting Author unless you are acting as an employee on behalf of your employer or a postgraduate student of an affiliated institution which is paying any applicable article publishing charge ("APC") for Open Access articles. Where the Submitting Author wishes to make the Work available on an Open Access basis (and intends to pay the relevant APC), the terms of reuse of such Open Access shall be governed by a Creative Commons licence – details of these licences and which Creative Commons licence will apply to this Work are set out in our licence referred to above.

Other than as permitted in any relevant BMJ Author's Self Archiving Policies, I confirm this Work has not been accepted for publication elsewhere, is not being considered for publication elsewhere and does not duplicate material already published. I confirm all authors consent to publication of this Work and authorise the granting of this licence.

For peer review only

Exploring the barriers to, and importance of, participant diversity in early phase clinical trials: an interview-based qualitative study of professionals and patient and public representatives

Robin Chatters (r.chatters@sheffield.ac.uk), Sheffield Clinical Trials Research Unit, Sheffield Centre for Health and Related Research, University of Sheffield, Sheffield, UK

Munya Dimairo (m.dimairo@sheffield.ac.uk), Sheffield Clinical Trials Research Unit, Sheffield Centre for Health and Related Research, University of Sheffield, Sheffield, UK

Cindy Cooper (c.l.cooper@sheffield.ac.uk), Sheffield Clinical Trials Research Unit, Sheffield Centre for Health and Related Research, University of Sheffield, Sheffield, UK

Shamila Ditta (s.ditta@sheffield.ac.uk), Sheffield Clinical Trials Research Unit, Sheffield Centre for Health and Related Research, University of Sheffield, Sheffield, UK

Jonathan Woodward (jonathan.woodward@sheffield.ac.uk), Sheffield Clinical Trials Research Unit, Sheffield Centre for Health and Related Research, University of Sheffield, Sheffield, UK

Katie Biggs (c.e.biggs@sheffield.ac.uk), Sheffield Clinical Trials Research Unit, Sheffield Centre for Health and Related Research, University of Sheffield, Sheffield, UK

Della Ogunleye (ogunleyedella5@gmail.com) (patient representative), London, UK

Fiona Thistlethwaite (fiona.thistlethwaite@nhs.net), Christie NHS Foundation Trust and University of Manchester, Manchester, UK

Christina Yap (christina.yap@icr.ac.uk), Clinical Trials and Statistics Unit, Institute of Cancer Research, Sutton, UK

Alexander Rothman (a.rothman@sheffield.ac.uk), Department of Infection, Immunity, and Cardiovascular Disease, Medical School, University of Sheffield and Sheffield Teaching Hospitals NHS Foundation Trust, Sheffield, UK

Correspondence to:

Robin Chatters, Sheffield Clinical Trials Research Unit, Sheffield Centre for Health and Related Research, The University of Sheffield, Regent Court, Regent Street, S1 4DA

For peer review only - http://bmjopen.bmj.com/site/about/guidelines.xhtml

r.chatters@sheffield.ac.uk

Keywords: Equality, diversity, inclusion, early phase trial, qualitative

Word count: 5708

Abstract

Objectives: To explore the importance of, and barriers to achieving, diversity in early phase clinical trials.

Design: Qualitative interviews analysed using thematic analysis.

Setting and participants: Five professionals (clinical researchers and methodologists) and three patient and public representatives (those with experience of early phase clinical trials and/or those from ethnic minority backgrounds) were interviewed between June and August 2022. Participants were identified via their institutional webpage, existing contacts or social media (e.g., X, formerly known as Twitter).

Results: Professionals viewed that diversity is not currently considered in all early phase clinical trials but felt that it should always be taken into account. Such trials are primarily undertaken at a small number of centres, thus limiting the populations they can access. Referrals from clinicians based in the community may increase diversity, however, those referred are often not from underserved groups. Referrals may be hindered by the extra resources required to approach and recruit underserved groups and participants often having to undertake "self-driven" referrals. Patient and public representatives stated that diversity is important in research staff and that potential participants should be informed of the need for diversity. Those from underserved groups may require clarification regarding the potential harms of a treatment, even if these are unknown. Education may improve awareness and perception of early phase clinical trials. We provide fourteen recommendations to improve diversity in early phase clinical trials.

Conclusions: Diversity should be considered in all early phase trials. Consideration is required regarding the extent of diversity and how it is addressed. The increased resources needed to recruit those from underserved groups may warrant funders to increase the funds to support the recruitment of such participants. The potential harms and societal benefits of the research should be presented to potential participants in a balanced but accurate way to increase transparency.

For peer review only - http://bmjopen.bmj.com/site/about/guidelines.xhtml

Strengths and limitations of this study

- We qualitatively interviewed a diverse sample of five professionals (clinical researchers and methodologists) and three patient representatives to explore the importance of, and barriers to achieving, diversity in early phase clinical trials.
- Data saturation was not achieved due to a small sample size; rather, we looked to achieve 'information power', as conceived by Malterud *et al.,* where the size of the study was determined by the amount of information the sample holds.
- Interviewees were predominately based in the UK and had experience of cancer early phase clinical trials—this therefore limited the conclusions that can be made regarding other disease areas, sectors and countries.

INTRODUCTION

The importance of equality, diversity and inclusion in clinical research is well documented, with the COVID-19 pandemic bringing into sharp focus the lack of inclusion of underserved groups in clinical trials [1–5]. The value of inclusion of such populations in late phase trials (i.e., in phases 3 and 4) is widely accepted, as treatments may affect diverse subsets of the population differently [6]. As such, there is guidance for researchers to consider groups routinely underserved in research when designing and conducting clinical trials [7–9]. There is evidence to suggest that diversity in such trials may be increased by improving staff competency through training and increased recruitment of staff from diverse backgrounds [6].

There is a lack of guidance regarding the importance of diversity and how to incorporate underserved groups into early phase clinical trials (EPCTs). Two reviews and a Food and Drug Administration (FDA) white paper provide guidance around increasing clinical trial diversity in the drug development process, but all lack guidance specific to EPCTs [10–12]. EPCTs are conducted in healthy volunteers or participants with a specific health condition and help us understand the feasibility of the therapeutic approach, the appropriate dose of the new treatment, how the drug should be administered, how the body responds to the treatment, and side effects. More so, they hugely inform the design and conduct of related later phase trials. Due to their small size and often high-risk nature, EPCTs have specific challenges regarding the inclusion of diverse populations. Despite the unknowns, the current lack of diversity in EPCTs is clear. EPCTs underrepresent women [13], older adults [14–16], those with lower education levels [17], those who live further away from

For peer review only - http://bmjopen.bmj.com/site/about/guidelines.xhtml

hospitals [17], those from deprived areas [18], ethnic minorities [19,20] and, for those studies undertaken in majority English speaking societies, individuals with limited English proficiency [21]. It has been shown that participants experiencing socioeconomic deprivation have worse outcomes in cancer trials than those not experiencing socioeconomic deprivation [22], however, we do not know if this is the case for EPCTs due to the lack of data on diversity. The development and deployment of COVID-19 vaccines highlighted the role of medical mistrust in preventing individuals from participating in EPCTs, especially in those from ethnically diverse communities [23,24].

There are a lack of interventions to address the inequalities within EPCTs [20]. Several studies have been undertaken to assess the barriers to participating in EPCTs, which include the information provided to potential participants [25], expectations of a therapeutic benefit from the trial treatment [26,27], lack of social and financial support, [28] and restrictive eligibility criteria [29]. However, these studies focus on general recruitment barriers, rather than those that prevent underserved populations from participating. Potential methods of increasing diversity include community outreach, increased diversity of staff, culturally tailored interventions, bilingual study teams [6,30,31], and helping elderly adults navigate barriers to enrolment [32].

Previous research has not discussed the importance of the inclusion of underserved populations in EPCTs, or when it may be important. It may be the case, for certain conditions, treatments, or contexts, that a focus on the representation of the population is less important and therefore diverse populations may not be required. For example, due to suspected homogeneity in how individuals may react to the treatment under investigation. However, excluding certain populations from such trials may lead to an inequitable health policy stacked against underrepresented subpopulations. There is also evidence of a potential 'trial effect' of improved outcomes, greater adherence to clinical guidelines and evidence-based practice of clinicians and institutions that take part in trials [33]. However, the consequences for patient health are uncertain.

We therefore undertook a qualitative study of professionals with knowledge of EPCTs (i.e., clinical researchers and methodologists) and patient and public representatives to explore in which situations diversity may be important in EPCTs, how diversity has been (and could be) incorporated into EPCTs, and the barriers to inclusion in such trials.

For peer review only - http://bmjopen.bmj.com/site/about/guidelines.xhtml

METHODS

Scope and participant eligibility

We undertook semi-structured qualitative interviews with both professionals (i.e., clinical researchers and methodologists with knowledge of EPCTs) and patient and public representatives (those with experience of EPCTs and/or those from ethnic minority backgrounds). Interviews with professionals explored the level of importance of diversity in EPCTs and when it may be appropriate, their experience of incorporating diversity into EPCTs, and the challenges of doing so. Interviews with the patient and public representatives focussed on the challenges of participating in EPCTs, and methods to improve diversity in such trials.

Recruitment of interview participants

Potential participants were identified by either searching institutional webpages or registries for individuals that are involved in EPCTs (e.g., clinical trials units (CTUs) that are listed on the UK Clinical Research Collaboration (UKCRC) CTU website as having expertise in phase 1 studies); asking study collaborators and other contacts for any individuals they are aware of who may be experienced in conducting EPCTs; and via social media (e.g., X, formerly known as Twitter).

Potential participants were sent an email with a participant information sheet (PIS) inviting them to participate in the study. To identify a purposive selection of participants for the interview, individuals were asked to complete a short survey to express their interest in the study which was administered using Qualtrics. The questionnaire requested key demographic data including location, age, ethnicity (patient and public representatives only), experience (professional or patient and public representative), clinical speciality (professionals only), area of expertise (professionals only), and gender. At the end of the survey, respondents were able to indicate whether they were interested in participating in an interview.

Participants were selected purposefully from those that completed the survey (n=22), maximising variation in key characteristics (area of expertise, ethnicity for the patient and public representatives only, years of experience, and gender). Additional participants were approached via email for the interview without having to complete the survey first if they were thought to increase the diversity of the sample.

Research ethics and participant consent process

Consent to participate in the semi-structured interviews was obtained via a consent form, which was completed by the participant prior to the interview, and after completing the survey (if applicable). The participant signed the consent form using an electronic (typed, or image of their signature

For peer review only - http://bmjopen.bmj.com/site/about/guidelines.xhtml

inserted into the form) signature. The form was then countersigned by the researcher, and a copy of the completed consent form was emailed back to the participant.

Sample size

Data saturation was not achieved due to limitations in funding. Rather, we looked to achieve 'information power', as conceived by Malterud *et al.* [34], where the size of the study was determined by the amount of information the sample holds, with studies encompassing narrow aims, dense specificity and strong dialogue having higher information power. We aimed to interview between eight and ten individuals.

Study design and data collection

The study used a phenomenological framework, as interviewees had direct experience of the phenomenon under study. Two semi-structured topic guides (see *supplementary materials*) were used (one for professionals and another for the patient and public representatives). These were developed by RC, with the aims of the study used as a starting point. A literature review was undertaken to inform the topic guide, identifying a lack of guidance specifically relating to EPCTs. As a result, literature regarding EDI in later phase trials (III or IV) was used to inform the interview questions [4,6,35]. The topic guides were reviewed by members of the study team, including DO (a patient and public representative), but they were not piloted. The topic guide was iteratively modified after each interview. Seven of the eight interviews were conducted by RC (a male Research Fellow with a BSc), with assistance from JW (a male Research Assistant with an MSc) and SD (a female Research Assistant with an MA). One interview was conducted by SD and JW. All three interviewers had the experience of working on clinical trials within a CTU, had previous experience of qualitative interviewing, and had a general interest in EDI in clinical research. Repeat interviews were not carried out, and transcripts were not returned to the participant for comment or correction. There were no other individuals present at the interviews and interviews lasted from 42 to 62 minutes. Participants undertook the interviewees either whilst at work or home. Field notes were not taken.

All interviews were undertaken via Google Meet, with the audio and video from the interview recorded (with consent) using in-built functionality within the Google Meet platform and transcribed for in-depth analysis. Transcripts were anonymised before analysis.

Emphasis was placed on collecting detailed data from experienced participants. Participants did not provide feedback on the findings. Patient and public interviewees were offered £50 as remuneration for their time.

For peer review only - http://bmjopen.bmj.com/site/about/guidelines.xhtml

Relationships of interviewee and interviewers

For most interviewees, there was no prior relationship between the interviewees and the interviewers. However, interviewees would have been aware of the interviewer's interest in this research area. Two interviewees (DO and FT) were involved in the study as collaborators. They provided input into the study protocol and topic guides and were therefore more aware than the other participants of the background, rationale and aims of the study. However, they were not involved in the analysis and only provided feedback on the final written manuscript.

Patient and public involvement

DO (a patient representative with experience of participating in research studies) provided input into all aspects of the study, including the aims and objectives, semi-structured topic guides, and findings.

Analysis

Data were analysed using thematic analysis, as described by Braun and Clarke [36]. NVivo software (version 12) was used to manage the data. RC and JW read two transcripts in order to familiarise themselves with their contents, then proceeding to independently generate codes through semantic analysis, deriving the themes from the data. Both researchers independently generated themes that related to the research questions and aims. RC and JW met to discuss the themes in order to ensure homogeneity. RC coded the remaining transcripts, adapting the codes where necessary. There were two separate coding trees for the two main participant types – professionals and patient and public representatives, with main themes including the barriers to incorporating diverse populations, the need for diversity in EPCTs, current practice and issues, thoughts on how to improve diversity, and any positive and negative effects of including diverse populations. The two coding trees were combined within the final analysis for readability as they included similar and complementing views on the same topics.

RESULTS

Study flow and characteristics of interviewees

Sixty-eight individuals, based across 31 academic, health-care and industry focussed institutions, were approached to participate in the study. Two individuals stated they did not want to take part due to a lack of time and five because of a lack of experience of EPCTs, as well as 53 who did not reply. Five professionals (based within the National Health Service (NHS) and at UK Universities), and three patient and public representatives were interviewed – their characteristics are presented in Table 1. All participants, except one patient and public representative, were based in the UK and had the experience of cancer EPCTs.

For peer review only - http://bmjopen.bmj.com/site/about/guidelines.xhtml

Table 1. Participant characteristics

Participant ID	Gender	Ethnicity*	Age range (years)	Role	Years of experience of early phase trials+	Clinical area of experience
Participant 1	Male	Not collected	25- 34	Professional (Operational Lead at a CTU)	6-10	Cancer
Participant 2	Female	Not collected	45-54	Professional (Doctor)	>16	Cancer
Participant 3	Female	Black African	55-64	Patient and public representative	Not collected	Cancer
Participant 4	Female	Not collected	45-54	Professional (Statistician)	11-15	Cancer
Participant 5	Male	Not collected	35-44	Professional (Statistician)	6-10	Cancer
Participant 6	Female	British Chinese	65-74	Patient and public representative	Not collected	Cancer
Participant 7	Female	White Canadian	35-44	Patient and public representative	Not collected	Neurological
Participant 8	Female	Not collected	35-44	Professional (Nurse)	11-15	Cancer

*Ethnicity only collected for the patient and public representatives.

+Only collected for professional participants.

CTU, clinical trials unit.

Views on current practice

Lack of focus on recruiting underserved groups

Professional interviewees stated that they do not currently specifically focus on trying to recruit underserved groups to EPCTs.

For peer review only - http://bmjopen.bmj.com/site/about/guidelines.xhtml

"I don't think it's something we consciously do. We basically open it to everyone who can enter into the trial, but we do not actively go and look for underserved patients." (Participant 4, professional)

Those that did recall conversations on increasing diversity described rudimentary discussions around ensuring variation in the location of sites in the UK (e.g., North or South), and providing translation services, rather than in-depth conversations around how to increase the diversity of their sample.

"I think most of the time [discussions around diversity] will be about geography. It will be about "We've got a centre in London and I think I know someone up in Manchester. That'll be good because then we've got somewhere up north." (Participant 5, professional)

Lack of diversity

It was evident that there is a lack of diversity in EPCTs, with three professionals describing the demographics of those that participate in EPCTs as being generally white, educated, affluent, and young.

"*We know from our work here that patients coming through to our phase one trials are usually from the largely white, largely middle-class population*." (Participant 2, professional)

Awareness of need for diversity in EPCTs

Participants felt that awareness is increasing, with three interviewees discussing studies they are aware of looking at the level of diversity in EPCTs, and another participant recently becoming aware of a funder requesting information about diversity in grant applications.

"The [EPCT] grant application I'm doing at the moment has three questions on [diversity], which we never had before. So, we're having to think about how we are going to address some of these issues." (Participant 1, professional)

The level of importance for diversity in EPCTs

All interviewees stated that the inclusion of underserved groups is important and should always be considered when undertaking EPCTs.

"*You'd never want to be ignoring [diversity in EPCTs], I don't think. Trying to think on the spot why you would – I can't think of an argument why you'd not want to consider it."* (Participant 1, professional)

Participants thought that there is a general need to increase diversity in EPCTs without specifically focusing on particular attributes, underserved groups, or by setting a quota for underserved groups in the protocol.

"I don't think you can start to tease out who you should and shouldn't exclude in a phase 1 trial, so we should include everyone." (Participant 2, professional)

For peer review only - http://bmjopen.bmj.com/site/about/guidelines.xhtml

"I don't know if working on a quota system of, say, "We're recruiting 30 participants and we need, in this particular study, we need at least 5 to be black" or "We want a 50:50 split male and female". I don't know how practical that is in the early phase setting." (Participant 5, professional)

There were no situations when ensuring diversity may not be important. Even when undertaking an EPCT in a specific genetic biomarker or disease (e.g., prostate cancer in men), it would still be important to seek diversity in other characteristics (e.g., socioeconomic status).

"[a] prostate cancer study obviously can only recruit males. If you wanted to look at an effect on a certain group or you did want to target a certain socioeconomic group then that could be an area where you'd target and exclude others." (Participant 1, professional)

The need for diversity in EPCTs

Interviewees discussed diversity in EPCTs in terms of the need for exploring treatment signals in diverse populations, research equity, and informing robust design and conduct of later phase trials.

Exploring treatment signals in diverse populations

The most frequently mentioned reason for increasing diversity in EPCTs was to ensure that EPCTs identify side-effects, tolerability, and efficacy in diverse populations early, as different populations may react variably to the study treatment. Many interviewees stated the aim of including diverse populations is to get a "feel" for the toxicities or a "signal" of the efficacy in these populations.

"… in the early phase you're not going to get the statistics to demonstrate that this drug is what the additional toxicities are, or that a different dose should be given or anything like that. I think it's about getting a feel for what the broad categories of toxicities are and what the dose is." (Participant 2, professional)

Improve research equity

A key reason for diversifying clinical trial populations is to improve fairness and to enable underserved groups to access novel treatments.

"It's not fair that there are marginalised groups that are missing out on an opportunity to explore clinical trials and it might be that it's not the right thing for them and that might be the conclusion, but I'd much rather that patient have that informed decision about that before they make that decision." (Participant 8, professional)

Interviewees explained that incorporating diverse groups into EPCTs allows treatments to be identified that may only be efficacious in certain populations. Assuming that treatments that are not efficacious in a Caucasian population do not work for everyone is an injustice if the treatment in fact works in other populations.

For peer review only - http://bmjopen.bmj.com/site/about/guidelines.xhtml

"[If you undertake a] trial [of] a drug in a Caucasian population and it doesn't have any efficacy signals […] there's an injustice there for the underserved ethnic populations, where actually it could be an efficacious drug." (Participant 2, professional)

Informing robust design and conduct of later phase trials

Improving the design and conduct of future trials was given as a reason to improve diversity, with specific considerations including understanding adherence to the new treatment in other populations, understanding how to better collect trial outcomes across diverse populations, and identifying stratification variables for later phase trials.

Barriers to diversity and methods to improve diversity

Interviewees provided their views on the barriers to incorporating diversity, and associated methods to improve representation, across the following themes: the location of recruiting centres, recruitment practices, the extra resources needed to recruit underserved groups, participant perception (awareness and transparency), and a lack of information on diversity in EPCTs. A summary of the proposed barriers and associated resolutions is provided in Table 2.

Location of recruiting centres in major cities

A main barrier to recruiting diverse populations was seen as the location of centres that undertake EPCTs, which are usually located in the centre of major cities. This can result in only those individuals who can travel to the centre being able to participate in the trial. This is compounded by the intensive time commitment required from participants to participate in these trials (e.g., intensive follow-up schedules).

"I think the main challenge for early phase is actually the fact that it's a lot more intensive if you enter into a trial in the early phase setting, compared to later. If you look at the time schedule of how many times you have to go into clinics, there's a lot more of them in the early phase." (Participant 4, professional)

Two methods were suggested to improve access to the research site. Firstly, both professionals and patient and public representatives suggested that participants and their carers or companions may need to be remunerated for their time (i.e., lost earnings), subsistence, and travel costs.

Secondly, referrals may be made from community hospitals. However, several participants stated that referrals from such centres often do not result in the recruitment of those from underserved groups due to participants often having to initiate the discussion regarding participation in an EPCT. To do so, participants require courage and conviction to ask their doctor about the trial, which may be less likely to be undertaken by those from underserved groups.

"Most of our patients that come to our unit are self-driven referrals [and] are well educated,

For peer review only - http://bmjopen.bmj.com/site/about/guidelines.xhtml

articulate, confident, and would question their consultant and are driven by their own research into clinical trials and what's available and are driving their referral, …But what it does mean is that I feel we are not getting a representative population to even consider a clinical trial." (Participant 8, professional)

These actions were felt to improve access to the research site, but not the issue of the time commitment required of participants, an issue to which interviewees did not provide a resolution.

"Even if you fund the travel, it's still difficult to have the time [to participate in an EPCT]." (Participant 4, professional)

Unnecessary eligibility criteria

The trial's eligibility criteria may indirectly reduce the diversity of the trial sample, which may be resolved by a patient or diversity representative reviewing the criteria to check for any elements that may inadvertently exclude individuals.

"It might be that you're excluding people who have a certain risk of another particular condition, whether that's a problem with their heart or some other issue … but what you're actually doing is you're making it far more likely that black women would not be eligible for that study because they're more likely to have that particular condition. Prior to the trial we engaged with an EDI representative to think about how we make this project as inclusive as possible. These were the steps we took. Therefore, our inclusion and exclusion criteria now read this, instead of this." (Participant 5, professional)

Participants need to be able to ask questions

Patient and public representatives felt it was important for the participant to be able to ask questions during the recruitment process. However, one professional participant stated many participants often do not feel able to do this, with clinicians also not encouraging questions due to a lack of time. One participant suggested that a sheet of prompts could be given to the participant to refer to during a consultation with their doctor to assist them in asking questions related to the EPCT.

"I developed a question prompt list for patients to have in their hand and they could then refer to the questions that I developed with patients about what they felt they perhaps should have asked at the beginning. it gave the patient permission to ask those questions, [for example] emotive questions like "Will this trial cure my cancer?" (Participant 8, professional)

For peer review only - http://bmjopen.bmj.com/site/about/guidelines.xhtml

For peer review only

Extra resources are needed to recruit underserved groups

Approaching underserved groups to participate in EPCTs was seen as time-consuming. The use of translators to help those without English as their first language to comprehend the trial was seen as a key issue, with this adding to the cost and time demands.

> *"Clinic appointments tend to take longer if there's an interpreter to make sure everything's been explained clearly and that the patient has a good understanding. And ultimately, if a clinic appointment takes longer and you have a high proportion of patients requiring interpreters for example, it potentially reduces the number of patients that you can recruit to a trial."* (Participant 2, professional)

These issues may be intensified in deprived areas due to increased staff turnover and the reliance on locum doctors who lack the time or training to undertake recruitment processes. The increased workload required to recruit those from underserved populations does not result in increased funding.

> *"Someone who's going to bring in patients from more diverse populations isn't going to generate any additional income, because we don't have a supply issue. And actually… [it] might decrease our income because patients who need a translator take longer in clinical time and might be able to treat fewer patients."* (Participant 2, professional)

Therefore, interviewees recommended changes that funders could make, including providing trial teams with extended trial recruitment phases to allow time to recruit those from underserved populations. Additionally, extra per-participant funding for recruiting those from underserved groups may be required.

> *"If you go down to the health economics of it, you've got to have a driver that will benefit us in some way and maybe that's some kind of funding from [the] NIHR, where if a patient is labelled as being from a hard-to-reach population, that patient attracts additional income."* (Participant 2, professional)

Awareness and perception of EPCTs

Lack of awareness of EPCTs

Interviewees suggested that the awareness of EPCTs could be increased. This could be achieved by providing potential participants with access to databases that allow participants to be identified. For example, using a similar portal to the Experimental Cancer Medicine Centres (ECMC) trial finder for cancer EPCTs (which is currently only open to clinicians). Conversations about recruitment to EPCTs should be initiated as early as possible (ideally by the general practitioner, GP). Patient advocacy groups could be used to assist in reaching out to underserved patient groups in a balanced manner.

For peer review only - http://bmjopen.bmj.com/site/about/guidelines.xhtml

"I think it also requires quite a strong patient advocacy group of some sort that currently I don't think exists for EPCTs, which I think you need to reach out for those people to kind of reach out to people who don't normally think about entering into early phase trials. Kind of highlighting pros and cons, being very balanced." (Participant 4, professional)

Perception of EPCTs

Patient and public representatives focussed on their perception of EPCTs. The safety of participating in an EPCT was seen as one of the main barriers to the inclusion of underserved groups, with the perceived risk being high, which was potentially exacerbated by the feeling of being a "guinea pig".

"I think in the Chinese culture, and some others, they are normally equated as guinea pigs and you've got to be self-sacrificing, or prepared to be, before you think about something like this, something drastic might befall you. Which is rather scaremongering." (Participant 6, patient and public representative)

Several professional and patient and public representatives proposed that education may improve awareness and perception of EPCTs, with suggestions including the provision of general health education in schools, educating underserved groups, and educating refers in the need for diversity within EPCTs. However, there was a lack of suggestions on how to achieve this.

*"I think [education is] key. Educating referrers and somehow educating [underserved] groups about the potential for accessing clinical trials. And I don't know fully how that's going to happen, how we can do that effectively." (*Participant 8, professional*)*

Communicating the level of risk associated with participating in an EPCT was seen as key, with safety being a key concern for public representatives, who may want detailed information about what is already known about the drug.

Improving transparency

Transparency was seen as being particularly important. Several methods of increasing transparency were suggested, including providing potential participants with testimonials from other participants and describing the need for diversity to those from underserved groups (i.e., why they are seeking diverse populations to participate in the study, and how they are including such groups).

One participant suggested that researchers should be honest if there is a lack of evidence regarding the safety of a new treatment (e.g., a drug).

"If the researcher feels that "if I share with the potential participant how little actually I know, they will lose confidence." But I would argue that conversely, if you don't share with me what you know and what you don't know, and why am I involved, do I have any valid role

in this? Can I actually make a contribution in some areas? So, in fact, transparency and that openness is confidence enforcing." (Participant 6, patient and public representative)

The need for transparency also extended to what *is not likely* to happen to a participant in an EPCT. One patient and public representative described that underserved populations have a lack of awareness of EPCTs and may assume that there is a significant risk of death. Researchers should therefore clarify the likely adverse events the participant may experience, for example, clarifying that death or paralysis is very unlikely. This could take the form of describing how other people have reacted to the drug.

"Has somebody like me done it before, how did they react to it?... It's just reassuring me that it's gonna be okay, you know, you wouldn't be paralysed." (Participant 3, patient and public representative).

Lack of research staff diversity

A lack of diversity was seen as not only a problem in the research participants themselves, but also in the staff and researchers that undertake and run such studies.

"The more diverse the people conducting the clinical trials, the more diverse it is, the better you see people forthcoming. "Oh, yes, I want to be part of that" When you don't see people like you in it, you know, what effect would it have on me? So, I know we're getting there but, you know, we still need to do more." (Participant 3, patient and public representative)

Lack of information on diversity in EPCTs

Professional participants suggested that little is currently known about the current level of diversity within EPCTs, and therefore, postcode data could be collected for all participants screened for entry into an EPCT to identify if trials are being accessed by underserved groups. However, there may be ethical challenges in collecting such data for those participants who do not consent to participate in the trial.

A schema, depicting the themes and the associations between them, is provided in Figure 1.

DISCUSSION

In this qualitative study, we have identified that the diversity of underserved groups does not seem to be currently routinely considered in EPCTs, but it is always viewed as relevant. The main benefit of including diverse populations in EPCTs is the identification of early signals of harms and efficacy in diverse populations, including underserved subpopulations, and providing equitable opportunities for patients. Study teams should make a concerted effort to recruit those from underserved groups into their EPCT and should seek to minimise the numerous barriers that prevent such individuals from participating. The most notable barrier appeared to be that EPCTs are primarily undertaken at

For peer review only - http://bmjopen.bmj.com/site/about/guidelines.xhtml

a small number of specialist centres, predominately located within the centre of large cities, thus limiting the populations they can access. To increase diversity, these centres may need to rely on referrals from “feeder” centres located in the community. However, the extra resources it requires to approach and recruit underserved populations (e.g., language translation) can prevent clinicians from referring them. Patient and public representatives stated that trust and communication are important – those recruiting underserved groups to EPCTs should be transparent regarding the need for diversity in clinical trials, the potential harms of the treatment, and if these are not known, clarifying the likelihood of severe illness to ease their concerns. Education of both patients/public and clinical staff was felt to be key to increasing awareness and perception of EPCTs.

The strengths of this study are that both professionals and patient and public representatives were sought to obtain the views of two of the key stakeholders involved in EPCTs. Both sets of individuals were diverse, in terms of the role of professional participants (including clinicians, statisticians, and operational leads) and the ethnicity of the patient and public representatives. Due to funding limitations only a small number of interviews were undertaken. Additionally, the approvals necessary to interview staff in the National Health Service (NHS) were not sought due to time limitations, and therefore only those staff that also had contracts with a university were interviewed, thus limiting the sample to those NHS staff that also had academic posts. Those from other organisations (e.g., the pharmaceutical industry) were not interviewed. Additionally, all except one interviewee’s experience was primarily in UK-based cancer EPCTs, therefore limiting the conclusions that can be made regarding other disease areas and countries. Funding was not available to undertake additional recruitment to interview those with expertise outside of cancer trials research. There was no provision for those individuals who required assistance to converse in English, which prohibited those with limited English proficiency from participating in this study. Lastly, ethnicity data was collected for public and patient only, and therefore we are unable to report and reflect on the ethnicity of ‘professional’ participants.

This study supports the literature regarding the need to increase diversity in clinical trials [6,37]. Some of the barriers and methods to increase diversity have been reported previously across both early and late phase trials, including adequately funding participants’ time and travel costs [38], restrictive eligibility criteria [12], burdensome trial procedures [12], informing community oncologists of the trial to aid recruitment [32], and translation and language issues [30]. We have identified that, due to time constraints, those who refer to clinical trials may not approach those from underserved groups, which has been found in a previous study [39]. Our study adds to this literature by clarifying that diversity is important in all EPCTs, and by identifying additional barriers

For peer review only - http://bmjopen.bmj.com/site/about/guidelines.xhtml

that, as far as we are aware, have not been previously reported. These additional barriers concern the effect of the location of EPCT units on their ability to access underserved groups and the lack of referrals to these units of underserved groups from referrers in the community. Such referrals may be hindered by the need for participants to "self-drive" the referral, which may be a barrier given the lack of knowledge of EPCTs in underserved groups. We have also identified specific issues related to transparency, including informing the participant the reason for the need to focus on the recruitment of underserved groups. Interviewees also suggested that an indication of the level of risk associated with the EPCT is provided during the recruitment process, even if the effects of the drug are unknown. It is important to note that, as per Good Clinical Practice (GCP) guidelines, the risks of participating in research should always be communicated to patients in a culturally responsive way [40]. However, participants in this study desired an estimation of the risks of research even when these cannot be quantified, for example, reassurance that the drug under test is unlikely to lead to death or significant injury.

This study implies that those designing and recruiting to EPCTs should attempt to increase the diversity of their sample by referring to the fourteen principles outlined in Box 1. In this box, the authors have supplemented the findings from the qualitative study with their own reflections, which are provided in *italics*.

It should be noted that given that those participating in EPCTs may have optimistic expectations of the effect of participating in an EPCT [27], highlighting the harms of such trials may have a negative effect on recruitment, so researchers may also describe to participants the potential societal benefits of EPCTs highlighted in this study (e.g., improving the equity of research, exploring early treatment signals in diverse populations, and informing the robust design of future trials). Future research should focus on exploring the barriers and enablers to recruitment in a larger diverse sample of participants, including NHS staff, and by investigating how potential harms are communicated to participants, especially in the absence of quantifiable data. Overcoming the inherent barriers to undertaking clinical trials in novel treatments, which, due to their risk, often require participants having to regularly attend the hospital for safety checks, may be challenging but would open up EPCTs to patient groups who are currently unable to participate in such trials. These "remote" EPCTs have been broached, but are rarely undertaken in practice [41]. Additionally, educational packages could be developed to increase awareness and improve the perception of EPCTs in underserved groups – future research may focus on the content, audience, timing, and format of such packages.

For peer review only - http://bmjopen.bmj.com/site/about/guidelines.xhtml

Declarations

Contributors

RC, MD and CC devised the study. RC, SD and JW collected the data and undertook the analysis. RC wrote the first draft of the report. DO was a patient representative who provided input into the design of the study and the findings. All authors, including AR, CY, FT and KB, provided input into the design and implementation of the study, critically reviewed the draft, and provided approval for publication.

Competing interests

The authors declare that they have no competing interests.

Funding

The study was funded by Research England via the Participatory Research Fund (X/175529).

Data availability statement

The dataset is the in-depth interview transcripts. Access is restricted to the study team in accordance with the requirements of the ethical approval.

Ethical approval and consent to participate

The study received favourable ethics approval from the research ethics committee based at the School of Health and Related Research (ScHARR), within the University of Sheffield (reference number: 045712). Participants provided informed consent to participate in the study and for their quotations to be anonymously published.

Acknowledgements

We would like to thank the eight participants for their time in participating in the interviews and for sharing their valuable knowledge with us. We remember Dolapo (Della) Ogunleye, an integral member of this study team, who passed away before this manuscript was published. Her passion, insights and dedication to improving diversity and inclusion of participants in research to achieve equitable patient care enriched every aspect of this work.

For peer review only - http://bmjopen.bmj.com/site/about/guidelines.xhtml

References

1 Diversity Data Report. Published Online First: 2021.https://www.nihr.ac.uk/about-us/our-key-priorities/equality-diversity-and-inclusion/diversity-data-report.htm

2 UK government sets out bold vision for the future of clinical research delivery - GOV.UK. https://www.gov.uk/government/news/uk-government-sets-out-bold-vision-for-the-future-of-clinical-research-delivery (accessed 12 Jan 2022).

3 Inclusion of Women and Minorities as Participants in Research Involving Human Subjects | grants.nih.gov. https://grants.nih.gov/policy/inclusion/women-and-minorities.htm (accessed 12 Jan 2022).

4 Improving inclusion of under-served groups in clinical research: Guidance from INCLUDE project. https://www.nihr.ac.uk/documents/improving-inclusion-of-under-served-groups-in-clinical-research-guidance-from-include-project/25435 (accessed 12 Jan 2022).

5 UKRI equality diversity and inclusion strategy: draft for consultation – UKRI. https://www.ukri.org/publications/equality-diversity-and-inclusion-strategy-draft-for-consultation/ukri-equality-diversity-and-inclusion-strategy-draft-for-consultation/ (accessed 17 Jan 2022).

6 Bodicoat DH, Routen AC, Willis A, *et al.* Promoting inclusion in clinical trials—a rapid review of the literature and recommendations for action. *Trials* 2021;**22**. doi:10.1186/S13063-021-05849-7

7 Treweek S, Banister K, Bower P, *et al.* Developing the INCLUDE Ethnicity Framework—a tool to help trialists design trials that better reflect the communities they serve. *Trials* 2021;**22**:1–12. doi:10.1186/S13063-021-05276-8/TABLES/3

8 Jutlla K. INCREASING PARTICIPATION OF BLACK ASIAN AND MINORITY ETHNIC (BAME) GROUPS IN HEALTH AND SOCIAL CARE RESEARCH. 2018.

9 Equality Impact Assessment Guidance and Template. http://www.acas.org.uk/media/pdf/s/n/Acas_managers_guide_to_equality_assessments.pdf (accessed 31 Aug 2022).

10 Moore KT, Grundmann O, Iwuchukwu O, *et al.* The Importance of Diversity and Inclusion in Drug Development and Clinical Trial Conduct. *J Clin Pharmacol* 2022;**62**:1475–9. doi:10.1002/jcph.2151

For peer review only - http://bmjopen.bmj.com/site/about/guidelines.xhtml

11 Gross AS, Harry AC, Clifton CS, *et al.* Clinical trial diversity: An opportunity for improved insight into the determinants of variability in drug response. *Br J Clin Pharmacol* 2022;**88**:2700–17. doi:10.1111/bcp.15242

12 Enhancing the Diversity of Clinical Trial Populations — Eligibility Criteria, Enrollment Practices, and Trial Designs Guidance for Industry. 2020. https://www.fda.gov/media/127712/download

13 Pinnow E, Sharma P, Parekh A, *et al.* Increasing Participation of Women in Early Phase Clinical Trials Approved by the FDA. *Women's Heal Issues* 2009;**19**:89–93. doi:10.1016/j.whi.2008.09.009

14 Baldini C, Charton E, Schultz E, *et al.* Access to early-phase clinical trials in older patients with cancer in France: the EGALICAN-2 study. *ESMO Open* 2022;**7**:100468. doi:10.1016/J.ESMOOP.2022.100468

15 Khan KH, Yap TA, Ring A, *et al.* Phase I trial outcomes in older patients with advanced solid tumours. *Br J Cancer* 2016;**114**:262–8. doi:10.1038/bjc.2015.477

16 Mahipal A, Denson AC, Djulbegovic B, *et al.* Effect of Age on Clinical Outcomes in Phase 1 Trial Participants. *Cancer Control* 2015;**22**:235–41. doi:10.1177/107327481502200217

17 Gad KT, Johansen C, Duun-Henriksen AK, *et al.* Socioeconomic differences in referral to phase i cancer clinical trials: A danish matched cancer case-control study. *J Clin Oncol* 2019;**37**:1111–9. doi:10.1200/JCO.18.01983

18 Mohd Noor A, Sarker D, Vizor S, *et al.* Effect of Patient Socioeconomic Status on Access to Early-Phase Cancer Trials. *J Clin Oncol* 2013;**31**:224–30. doi:10.1200/JCO.2012.45.0999

19 Dunlop HM, Fitzpatrick E, Kurti K, *et al.* Abstract PR-05: Worsening disparities of racial minority participation in phase 1 early drug development trials in the United States, 2001-2018. *Cancer Epidemiol Biomarkers Prev* 2022;**31**:PR-05-PR-05. doi:10.1158/1538-7755.DISP21-PR-05

20 Perni S, Moy B, Nipp RD. Disparities in phase 1 cancer clinical trial enrollment. *Cancer* 2021;**127**:4464–9. doi:10.1002/cncr.33853

21 Willis A, Isaacs T, Khunti K. Improving diversity in research and trial participation: the challenges of language. *Lancet Public Heal* 2021;**6**:e445–6. doi:10.1016/S2468-2667(21)00100-6

For peer review only - http://bmjopen.bmj.com/site/about/guidelines.xhtml

22 Unger JM, Moseley AB, Cheung CK, *et al.* Persistent Disparity: Socioeconomic Deprivation and Cancer Outcomes in Patients Treated in Clinical Trials. *J Clin Oncol* 2021;**39**:1339–48. doi:10.1200/JCO.20.02602

23 Charura D, Hill AP, Etherson ME. COVID-19 Vaccine Hesitancy, Medical Mistrust, and Mattering in Ethnically Diverse Communities. *J Racial Ethn Heal Disparities* 2023;**10**:1518–25. doi:10.1007/s40615-022-01337-z

24 Thompson HS, Manning M, Mitchell J, *et al.* Factors Associated With Racial/Ethnic Group–Based Medical Mistrust and Perspectives on COVID-19 Vaccine Trial Participation and Vaccine Uptake in the US. *JAMA Netw Open* 2021;**4**:e2111629. doi:10.1001/jamanetworkopen.2021.11629

25 Cox K. Informed consent and decision-making: patients' experiences of the process of recruitment to phases I and II anti-cancer drug trials. *Patient Educ Couns* 2002;**46**:31–8. doi:10.1016/s0738-3991(01)00147-1

26 Meropol NJ, Weinfurt KP, Burnett CB, *et al.* Perceptions of patients and physicians regarding phase I cancer clinical trials: implications for physician-patient communication. *J Clin Oncol* 2003;**21**:2589–96. doi:10.1200/JCO.2003.10.072

27 Catt S, Langridge C, Fallowfield L, *et al.* Reasons given by patients for participating, or not, in Phase 1 cancer trials. *Eur J Cancer* 2011;**47**:1490–7. doi:10.1016/j.ejca.2011.02.020

28 Castillo G, Lalu MM, Asad S, *et al.* Navigating choice in the face of uncertainty: using a theory informed qualitative approach to identifying potential patient barriers and enablers to participating in an early phase chimeric antigen receptor T (CAR-T) cell therapy trial. *BMJ Open* 2021;**11**:e043929. doi:10.1136/bmjopen-2020-043929

29 Ho J, Pond GR, Newman C, *et al.* Barriers in phase I cancer clinical trials referrals and enrollment: five-year experience at the Princess Margaret Hospital. *BMC Cancer* 2006;**6**:263. doi:10.1186/1471-2407-6-263

30 Chalela P. Promoting Factors and Barriers to Participation in Early Phase Clinical Trials: Patients Perspectives. *J Community Med Health Educ* 2014;**04**. doi:10.4172/2161-0711.1000281

31 Pham B, Earle N, Rabel K, *et al.* Maximizing the diversity of participants in a phase II clinical trial of optical technologies to detect cervical neoplasia. *Gynecol Oncol* 2007;**107**:S208–14.

For peer review only - http://bmjopen.bmj.com/site/about/guidelines.xhtml

doi:10.1016/J.YGYNO.2007.07.063

32 Basche M, Barón AE, Eckhardt SG, *et al.* Barriers to Enrollment of Elderly Adults in Early-Phase Cancer Clinical Trials. *J Oncol Pract* 2008;**4**:162–8. doi:10.1200/JOP.0842001

33 Clarke M, Loudon K. Effects on patients of their healthcare practitioner's or institution's participation in clinical trials: a systematic review. *Trials* 2011;**12**:16. doi:10.1186/1745-6215-12-16

34 Malterud K, Siersma VD, Guassora AD. Sample Size in Qualitative Interview Studies. *Qual Health Res* 2016;**26**:1753–60. doi:10.1177/1049732315617444

35 Ford JG, Howerton MW, Lai GY, *et al.* Barriers to recruiting underrepresented populations to cancer clinical trials: A systematic review. *Cancer* 2008;**112**:228–42. doi:10.1002/CNCR.23157

36 Braun, V., Clark V. Using thematic analysis in psychology. *Qual Res Psychol* 2006;**3**:77–101.

37 Inclusion of Older Adults in Cancer Clinical Trials Guidance for Industry. https://www.fda.gov/media/156616/download#:~:text=The issue of older adults being underrepresented in,malignancy%2C all of which may increase with age.

38 Bierer BE, White SA, Gelinas L, *et al.* Fair payment and just benefits to enhance diversity in clinical research. *J Clin Transl Sci* 2021;**5**:e159. doi:10.1017/cts.2021.816

39 Gomes F, Descamps T, Lowe J, *et al.* Enrolment of older adults with cancer in early phase clinical trials—an observational study on the experience in the north west of England. *Age Ageing* 2021;**50**:1736–43. doi:10.1093/ageing/afab091

40 Guideline for good medical practice E6(R2). https://www.ema.europa.eu/en/documents/scientific-guideline/ich-guideline-good-clinical-practice-e6r2-step-5_en.pdf

41 Alemayehu D, Hemmings R, Natarajan K, *et al.* Perspectives on Virtual (Remote) Clinical Trials as the "New Normal" to Accelerate Drug Development. *Clin Pharmacol Ther* 2022;**111**:373–81. doi:10.1002/cpt.2248

For peer review only - http://bmjopen.bmj.com/site/about/guidelines.xhtml

FIGURE TITLE

Figure 1. Thematic schema

Table 2. Barriers and facilitators to increasing diversity in EPCTs

**Category**	**Barrier**	**Facilitator**
Patient factors	EPCTs require intensive time commitment from trial participants, which may deter involvement	None discussed
	Participants may have to undertake "self-driven" referrals and require the motivation and courage to initiate discussions regarding EPCTs with their clinician	• Educate potential participants regarding the EPCTs that are available to them<br>• Educate clinicians to enable them to approach and recruit participants to EPCTs
	A perceived lack of transparency may result in those from underserved groups being unlikely to participate in an EPCT	• Provide potential participants with testimonials from other participants<br>• Describe the need for diversity in the EPCT to those from underserved groups and the importance of participating
	It is important for participants to ask questions about the EPCT when discussing their potential participation, but participants may not ask questions, and clinicians may not encourage them, due to a lack of time	• A list of potential questions can be given to participants to prompt them during a consultation with their doctor/clinician
	There is a lack of awareness of EPCTs amongst underserved groups	• Discussions around participating in an EPCT should be initiated as early as possible, ideally by the GP

For peer review only - http://bmjopen.bmj.com/site/about/guidelines.xhtml

		• Education to improve awareness, both for patients and clinical staff.<br>• Permit use of trial databases to allow participants to locate EPCTs (e.g., Experimental Cancer Medicine Centres (ECMC) in cancer).<br>• Patient advocacy groups could be established to assist in relaying information to underserved groups in a balanced manner.
	Those from underserved groups may have concerns around the safety of participating in an EPCT	• Communicate the level of risk associated with the EPCT with the participant<br>• Provide detailed information about what is already known about study treatment<br>• Provide detail as to what is unlikely to happen to participants (e.g., death, paralysis)<br>• Educate patients to help improve their perception of EPCTs.
Patient factors / trial design and implementation	Many centres that recruit to EPCTs are located in the centre of major cities which may be challenging for certain populations to access	• Referrals can be made from community hospitals<br>• Provide reimbursement to trial participants and their carers for travel and subsistence costs
Trial design and implementation	A lack of diversity in trial staff and researchers may deter participants from taking part	• Increase the diversity of staff that undertake and run EPCTs
	Eligibility criteria may inadvertently reduce the diversity of the trial sample	• Engage with a patient or diversity representative to review eligibility criteria

For peer review only

	Approaching underserved groups to participate in an EPCT requires extra resources	• Additional funding and time may be required to recruit those from underserved groups

Box 1. Recommendations for researchers and funders

**Recommendations for researchers**
1) Increasing the diversity of the sample should always be considered in EPCTs to a) explore treatment signals in diverse populations, b) improve research equity, and c) inform the robust design and conduct of later phase trials. *Some diseases have specific diversity profiles, which should be reflected in the trial sample.*
2) Those undertaking EPCTs should make a concerted effort to recruit those from underserved groups. The diversity of EPCTs should generally be increased, rather than focussing on specific attributes or quotas.
3) Important markers of diversity (e.g. postcode, *ethnicity, age*) should be consistently collected and report from all those that are screened to participate in EPCTs.
4) In order to reach diverse communities, potential participants may be referred from community hospitals. However, participants may still struggle to access the main trial site, so reimbursement for participants and their carers time and travel costs should be considered. *Enhancing participant awareness of the reimbursement of travel and other costs is important.*
5) Education of both participants and staff is important to ensure both parties are aware of EPCTs, in order to enable recruitment and retention.
6) Researchers should ensure eligibility criteria do not inadvertently exclude those from underserved groups and should seek input from patient or diversity representatives into these criteria. *However, this should be balanced with the risk of the EPCT – some exclusions may be justified based on safety concerns.*
7) A list of prompts may be provided to participants to refer to during conversations regarding participation in an EPCT to assist them in asking questions about the study.

For peer review only - http://bmjopen.bmj.com/site/about/guidelines.xhtml

8) Discussions around entry into an EPCT should commence as early as possible in the participant's journey, ideally with the GP.
9) Participants from underserved groups may be concerned about the risks of participating in an EPCT. In order to reassure them, researchers may:<br>a. Clearly communicate the level of risk associated with the EPCT with the participant. The societal benefits of participating in an EPCT may also be relayed to participants (see principle 1);<br>b. Provide detailed information about what is already known about the study treatment;<br>c. Provide an honest explanation of the amount available regarding the evidence regarding the effects of the study treatment;<br>d. Provide information as to what is *unlikely* to happen to participants as a result of the study treatment (e.g., death, paralysis).
10) In order to assist in relaying information to underserved groups in a balanced manner, patient advocacy groups may be formed.
11) Due to a perceived lack of transparency, those from underserved groups may be provided with testimonials from other participants. The need for diversity in the EPCT may be explained to such individuals.
12) The diversity of those staff that recruit to EPCTs should be increased in order to not deter underserved groups from participating.
**Recommendations for funders**
13) Funders may need to provide additional per participant funds for the recruitment of those from underserved groups due to the additional resources required to recruit such individuals.
14) Funders should request details of the methods investigators will use to identify, approach and recruit those from underserved groups in grant applications.

For peer review only

For peer review only - http://bmjopen.bmj.com/site/about/guidelines.xhtml

Figure 1: Thematic Schema

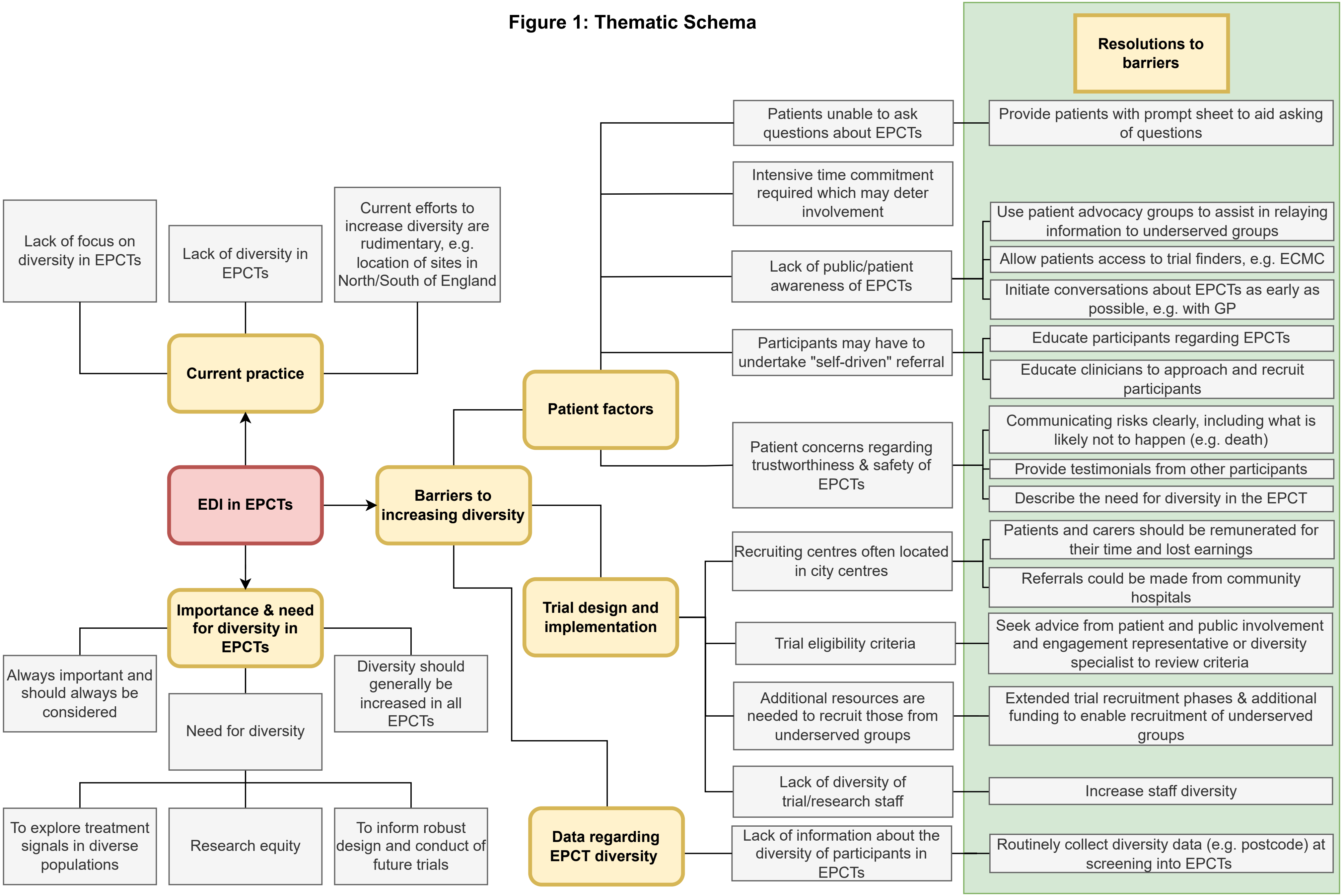

EDI in early phase trials – qualitative interview topic guide (patients and research participants)

NOTES: This topic guide is a flexible tool and may be revised as new areas of interest arise during the process of data collection. The wording of questions is for guidance only and can be varied to suit the natural style of the interviewer and the level of understanding of the participant.

---

1. Welcome and context setting:

- Introduce yourself
- Guide the participant through the Participant Information Sheet
- "The interview will last approximately 45 to 60 minutes"
- Explain that, "to help us with this study, we would like to make a recording of what we all say today, but nobody will be able to identify you from that recording other than me. Is that okay?"
- Guide the participant through the consent form
- "You are free to withdraw at any point during the interview and you don't have to answer any of the questions if you don't want to, but once the interview has finished I will be unable to remove your information from the study, if you chose to withdraw"
- Check they are happy to continue and ask if there are any questions.

2. Describe to the interviewee the definitions of underserved groups and EDI (in lay person language) we are using (these will also be provided to participants to refer to):

- Remind participant why we are doing this research
- Give brief definition of "EDI"
- Give brief definition of an early phase trial

3. Questions

QUESTION 1: What do you understand of early phase trials?

PROMPT: Have you been approached to take part in such a study?

QUESTION 2: What is your experience of the process of being approached to take part, or taking part in, early phase trials?

PROMPT: How were you approached to take part?
PROMPT: How did you find being approached?
PROMPT: How did you make the decision as to whether or not to take part?
PROMPT: What factors influenced your decision to either participate, or not participate?

QUESTION 3: What is your experience of taking part (or being approached to take part in) early phase trials?

PROMPT: What challenges were there to you participating in early phase trials?

For peer review only - http://bmjopen.bmj.com/site/about/guidelines.xhtml

Cultural and institutional barriers, attitudes and beliefs, emotional and psychological barriers, financial barriers

PROMPT: Were there any enablers?
PROMPT: What challenges are there for others who are from underserved groups?
PROMPT: anything else you would like to mention?

QUESTION 4: What influences your decision regarding whether or not to participate in early phase trials?

QUESTION 5: What enables you to take part in early phase trials?

PROMPT: What challenges are there to you participating in early phase trials?
PROMPT: What challenges are there for others who are from underserved groups?

QUESTION 6: What benefits are there to taking part in these trials?

PROMPT: What benefits are there to you participating in early phase trials?
PROMPT: What benefits are there for others who are from underserved groups?

QUESTION 7: What can be done to encourage more people to participate in early phase trials?

For peer review only

For peer review only - http://bmjopen.bmj.com/site/about/guidelines.xhtml

EDI in early phase trials – qualitative interview topic guide

NOTES: This topic guide is a flexible tool and may be revised as new areas of interest arise during the process of data collection. The wording of questions is for guidance only and can be varied to suit the natural style of the interviewer and the level of understanding of the participant.

1. Welcome and context setting:

- Introduce yourself
- Remind the participant of the aims of the study
- Guide the participant through the Participant Information Sheet
- "The interview will last approximately 45 to 60 minutes"
- Explain that, "to help us with this study, we would like to make a recording of what we all say today, but nobody will be able to identify you from that recording other than me. Is that okay?"
- Guide the participant through the consent form
- "You are free to withdraw at any point during the interview and you don't have to answer any of the questions if you don't want to, but once the interview has finished I will be unable to remove your information from the study, if you chose to withdraw"
- Check they are happy to continue and ask if there are any questions.

Remind the participants of the definition of underserved groups:

We are using the National Institute for Health Research's (NIHR's) guidance from the INnovations in CLinical trial design and delivery for the UnDEr-served (INCLUDE) project to define an underserved group, which states that:

> "The work of the NIHR-INCLUDE project shows that there is no single definition for an under-served group. Some key characteristics that are common to several under-served groups are:
>
> - Lower inclusion in research than one would expect from population estimates
> - High healthcare burden that is not matched by the volume of research designed for the group
> - Important differences in how a group responds to or engages with healthcare interventions compared to other groups, with research neglecting to address these factors
>
> "A key finding from our work is that the definition of 'under-served' is often very context and study specific. An under-served group for one disease or type of study may be the opposite to that of another."

EDI may be important due to:

- Lower inclusion of underserved groups in research than one would expect from population estimates

- High healthcare burden that is not matched by the volume of research designed for the group

For peer review only - http://bmjopen.bmj.com/site/about/guidelines.xhtml

- Important differences in how a group responds to or engages with healthcare interventions compared to other groups, with research neglecting to address these factors

2. Questions (remind participant to give examples, where appropriate)

QUESTION 1: Generally, what do you think is the value of including underserved groups in early phase trials?

PROMPT: Is there any value to including underserved groups in early phase trials?

PROMPT: Should early phase trials provide equal access to underserved groups (diversity)?

PROMPT: Should early phase trials recognise differences between individuals?

PROMPT: What are the positive consequences?

PROMPT: What are the negative consequences?

PROMPT: Is the value of underserved groups in early phase different from late phase and if so how?

QUESTION 2: When may the inclusion of underserved groups in early phase trials be warranted, and unwarranted (with reasons)?

PROMPT: Are there any situations (e.g. diseases, treatments) where underserved groups is required? If so, why?

PROMPT: Are there any situations (e.g. diseases, treatments) where underserved groups is less important? If so, why?

QUESTION 3: What is your view on the outcome of including underserved groups in early phase trials?

PROMPT: Does including (or not including) underserved groups affect future, later phase trials?

PROMPT: Does including (or not including) underserved groups affect the regulatory process?

QUESTION 4: Have you incorporated underserved groups into any early phase trials you have conducted?

PROMPT: Did you specifically plan any strategies to include individuals from underserved groups? Which groups?

PROMPT: Why did you (or why didn't you) look to include underserved groups in the trial?

QUESTION 5: How did you (OR HOW WOULD YOU) incorporate underserved groups into previous early phase trials?

PROMPT: How did you identify and approach those from underserved groups?

PROMPT: Were there any other design considerations taken into account? E.g. translation, reducing burden, amending exclusion criteria.

For peer review only - http://bmjopen.bmj.com/site/about/guidelines.xhtml

QUESTION 6 What are the challenges of including underserved groups in early phase trials? How have you, or would you, overcome these?

PROMPT: Have you encountered any challenges when incorporating underserved groups?

PROMPT: How did you, or might you overcome these challenges?

(if not already mentioned): Are there any challenges with regards to incorporating multiple underserved groups in a small early phase trial?

QUESTION 7: What enables (or helps) the incorporation of underserved groups into early phase trials?

Describe the definitions of underserved groups and EDI we are using (these will also be provided to participants to refer to). Ask participant to reflect on these definitions:

EDI:

Equality – Individuals are not treated less favourably due to their characteristics and have equal opportunities to take part in early phase trials, and are provided with equal opportunities if they decide to take part in a trial.

QUESTION: What are your reflections on this definition? Would you change it in any way?

QUESTION: What does equality mean in the context of early phase trials?

QUESTION: Do you think early phase trials provide equal access to groups (and do you think they should)?

QUESTION: Do you think people from particular groups are excluded from early phase trials?

Diversity – Recognising and respecting differences in individuals who are approached to take part, and decide to take part, in early phase trials.

QUESTION: What are your reflections on this definition? Would you change it in any way?

QUESTION: What does diversity mean in the context of early phase trials?

QUESTION: Do you think early phase trials recognise and respect the differences of individuals? Do you think they should?

Inclusion – Creating an environment where everyone feels welcomed and valued.

QUESTION: What does inclusion mean in the context of early phase trials?

QUESTION: Do you think early phase trials are inclusive? Do you think they should be?

For peer review only - http://bmjopen.bmj.com/site/about/guidelines.xhtml

COREQ (COnsolidated criteria for REporting Qualitative research) Checklist

A checklist of items that should be included in reports of qualitative research. You must report the page number in your manuscript where you consider each of the items listed in this checklist. If you have not included this information, either revise your manuscript accordingly before submitting or note N/A.

Topic	Item No.	Guide Questions/Description	Reported on Page No.
**Domain 1: Research team and reflexivity**			
Personal characteristics			
Interviewer/facilitator	1	Which author/s conducted the interview or focus group?	
Credentials	2	What were the researcher's credentials? E.g. PhD, MD	
Occupation	3	What was their occupation at the time of the study?	
Gender	4	Was the researcher male or female?	
Experience and training	5	What experience or training did the researcher have?	
Relationship with participants			
Relationship established	6	Was a relationship established prior to study commencement?	
Participant knowledge of the interviewer	7	What did the participants know about the researcher? e.g. personal goals, reasons for doing the research	
Interviewer characteristics	8	What characteristics were reported about the inter viewer/facilitator? e.g. Bias, assumptions, reasons and interests in the research topic	
**Domain 2: Study design**			
Theoretical framework			
Methodological orientation and Theory	9	What methodological orientation was stated to underpin the study? e.g. grounded theory, discourse analysis, ethnography, phenomenology, content analysis	
Participant selection			
Sampling	10	How were participants selected? e.g. purposive, convenience, consecutive, snowball	
Method of approach	11	How were participants approached? e.g. face-to-face, telephone, mail, email	
Sample size	12	How many participants were in the study?	
Non-participation	13	How many people refused to participate or dropped out? Reasons?	
Setting			
Setting of data collection	14	Where was the data collected? e.g. home, clinic, workplace	
Presence of non-participants	15	Was anyone else present besides the participants and researchers?	
Description of sample	16	What are the important characteristics of the sample? e.g. demographic data, date	
Data collection			
Interview guide	17	Were questions, prompts, guides provided by the authors? Was it pilot tested?	
Repeat interviews	18	Were repeat inter views carried out? If yes, how many?	
Audio/visual recording	19	Did the research use audio or visual recording to collect the data?	
Field notes	20	Were field notes made during and/or after the inter view or focus group?	
Duration	21	What was the duration of the inter views or focus group?	
Data saturation	22	Was data saturation discussed?	
Transcripts returned	23	Were transcripts returned to participants for comment and/or	

For peer review only

Topic	Item No.	Guide Questions/Description	Reported on Page No.
		correction?	
**Domain 3: analysis and findings**			
Data analysis			
Number of data coders	24	How many data coders coded the data?	
Description of the coding tree	25	Did authors provide a description of the coding tree?	
Derivation of themes	26	Were themes identified in advance or derived from the data?	
Software	27	What software, if applicable, was used to manage the data?	
Participant checking	28	Did participants provide feedback on the findings?	
Reporting			
Quotations presented	29	Were participant quotations presented to illustrate the themes/findings? Was each quotation identified? e.g. participant number	
Data and findings consistent	30	Was there consistency between the data presented and the findings?	
Clarity of major themes	31	Were major themes clearly presented in the findings?	
Clarity of minor themes	32	Is there a description of diverse cases or discussion of minor themes?	

Developed from: Tong A, Sainsbury P, Craig J. Consolidated criteria for reporting qualitative research (COREQ): a 32-item checklist for interviews and focus groups. *International Journal for Quality in Health Care*. 2007. Volume 19, Number 6: pp. 349 – 357

Once you have completed this checklist, please save a copy and upload it as part of your submission. DO NOT include this checklist as part of the main manuscript document. It must be uploaded as a separate file.

For peer review only
